# Drug delivery methods for cancer immunotherapy

**DOI:** 10.1007/s13346-023-01405-9

**Published:** 2023-08-16

**Authors:** Edgar Pérez-Herrero, Olivia L. Lanier, Neha Krishnan, Abby D’Andrea, Nicholas A. Peppas

**Affiliations:** 1 Departamento de Ingeniería Química y Tecnología Farmacéutica, Universidad de La Laguna, La Laguna, Tenerife, Spain; 2 Instituto Universitario de Tecnologías Biomédicas, Universidad de La Laguna, La Laguna, Tenerife, Spain; 3 Department of Biomedical Engineering, The University of Texas at Austin, Austin, TX USA; 4 Department of Chemical Engineering, The University of Texas at Austin, Austin, TX USA; 5 Institute for Biomaterials, Drug Delivery & Regenerative Medicine, The University of Texas at Austin, Austin, TX USA; 6 Division of Molecular Pharmaceutics and Drug Delivery, College of Pharmacy, The University of Texas at Austin, Austin, TX USA; 7 Department of Pediatrics, Dell Medical School, The University of Texas at Austin, Austin, TX USA; 8 Department of Surgery & Perioperative Care, Dell Medical School, The University of Texas at Austin, Austin, TX USA

**Keywords:** Immunotherapy, Cancer, Hematologic cancers, Drug delivery, Nanocarriers, Hydrogels

## Abstract

Despite the fact that numerous immunotherapy-based drugs have been approved by the FDA for the treatment of primary and metastatic tumors, only a small proportion of the population can benefit from them because of primary and acquired resistances. Moreover, the translation of immunotherapy from the bench to the clinical practice is being challenging because of the short half-lives of the involved molecules, the difficulties to accomplish their delivery to the target sites, and some serious adverse effects that are being associated with these approaches. The emergence of drug delivery vehicles in the field of immunotherapy is helping to overcome these difficulties and limitations and this review describes how, providing some illustrative examples. Moreover, this article provides an exhaustive review of the studies that have been published to date on the particular case of hematological cancers.

**Graphical Abstract:**

(Created with BioRender)

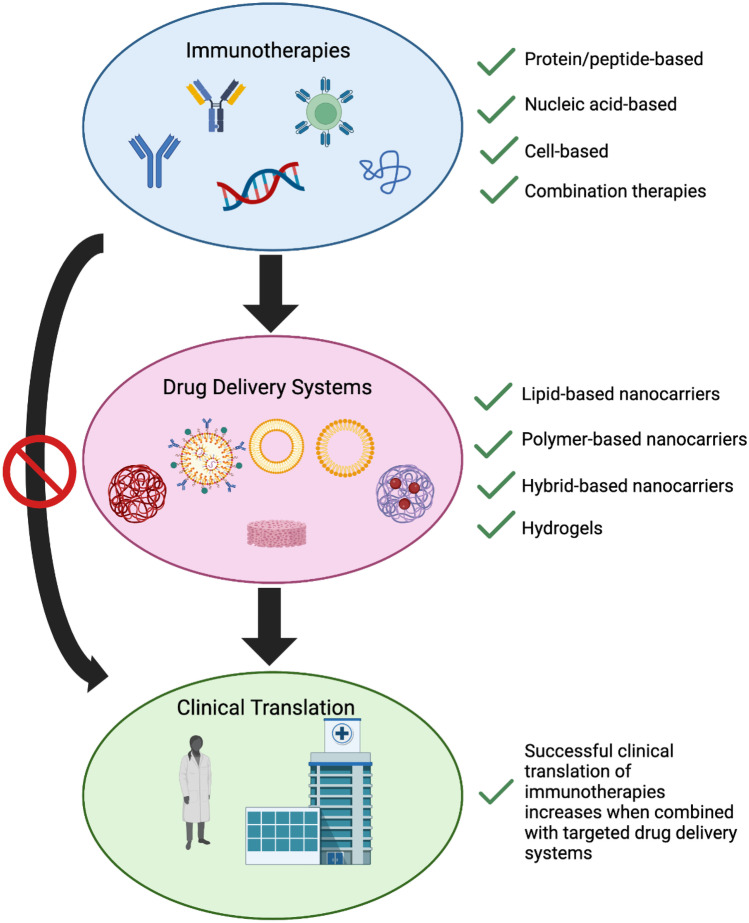

## Introduction

Immunotherapy has recently emerged in the cancer field as a promising and effective alternative to the conventional chemo- and radiotherapy and surgery to treat primary and metastatic tumors by using the innate and/or adaptive immune systems against them, obtaining long-term outcomes, the “immunological memory,” and leading to patient remissions [1]. In fact, currently, ten immune checkpoint inhibitors that target PD-1, PD-L1, CTLA-4, and LAG-3 have been approved by the Food and Drug Administration (FDA) to treat eighteen different tumors since the first approval of ipilimumab in 2011 [2–6]. Moreover, the FDA has approved one therapeutic cancer vaccine in 2010, sipuleucel-T (Provenge®), four cytokines with a mild clinical efficacy for solid and liquid tumors, one immune adjuvant, imiquimod, for the treatment of basal cell carcinoma, and six CAR (chimeric antigen receptor) T cell therapies between 2017 and 2022, being lisocabtagene maraleucel (Breyanzi®), idecabtagene vicleucel (Abecma®) and ciltacabtagene autoleucel (Carvykti®) the last to be approved on February 5 and March 26, 2021, and February 28, 2022 [6–11].

Currently, however, only a small number of cancer patients can benefit from immunotherapy due to *primary resistanc*e. Among those who initially responded, a relapse may occur due to an *acquired resistance* to immunotherapy [12]. The clinical translation of immunotherapy in the treatment of cancer is limited by numerous challenges. For example, the short circulatory half-lives of cytokines and their inefficient delivery to the target site lead to the use of high-dose therapies, which are associated with a significant systemic toxicity, immunosuppression, and immunogenicity [7]. Similarly, the targeting capacity of CAR T cell therapies may be reduced by tumor resistance events. Moreover, all FDA-approved CAR T cell therapies are indicated for the treatment of hematological cancers, as their application in solid tumors is very difficult due to the complications of specifically targeting the solid tumor antigens and entering into the tumor tissue [7, 13]. Likewise, immune checkpoint inhibitors have shown limited accumulations in the tumor site, due to their poor specificity, which can also affect normal tissues and produce serious autoimmune diseases [14]. Therefore, it is clear that there is a need to increase the clinical efficacy of immunotherapy by effective and appropriate drug delivery methods, vehicles, or mechanisms. Moreover, it should be noted that in the case of hematological cancers, the targeting and biodistribution limitations of these therapies make it mandatory for their vehiculization to improve their outcome and permit their translation to the clinical practice.

Drug delivery systems have the potential to target cancer cells directly, reducing off-target side effects and acquired resistance. They also decrease the required dose, leading to a reduction in toxicity, and can improve efficacy and allow the tunability of the immune response. Drug delivery vehicles can improve shelf-life, leading to scalable products, and can protect the therapy from degradation or recognition from the immune system when necessary. They can also be used to alter drug release rates which can lead to numerous advantages for patients [15]. For example, they can reduce the frequency of intravenous administration, thereby reducing patient discomfort and increasing compliance. They allow for a single dose to remain in the desired blood concentration range for extended time periods through release mechanisms such as diffusion from the carrier or degradation of the carrier. They also allow for drugs with short half-lives to have an increased lifetime within the patient’s blood stream by protecting them from degradation. Furthermore, they reduce hazardous peaks in concentration.

This review addresses the potential of lipid, polymer, and hybrid-based nanocarriers, as well as hydrogels and scaffolds, to revolutionize the field of cancer immunotherapy, paying special attention to the particular case of the treatment of hematological cancers since the abnormal vasculature, the stroma tissue, and the elevated intra-tumoral pressure in solid tumors limit the penetration and efficacy of immunotherapeutic drugs in their complex and immune-suppressive tumor microenvironment. A detailed description of the physiopathology, diagnosis, and current treatment options of the main hematological cancers can be found in the reference [6].

## Drug delivery approaches for immunotherapy in cancer

### Lipid-based nanocarriers used in immunotherapy treatment

#### Overview

Lipids, by their biomimetic nature, can cross different biological membranes and, due to their abundance in the organism, are considered non-toxic. Lipid-based nanocarriers, especially liposomes, include the main benefits that a nanocarrier can offer with those added by lipids, including low toxicity, high biodegradability and biocompatibility, and efficient cellular uptake. The capacity of liposomes to incorporate both hydrophilic and hydrophobic bioactive molecules in their inner hydrophilic cavity and inside their hydrophobic bilayer membrane, respectively, makes them a good option for the efficient delivery of immunotherapeutics to specific target sites [16]. Tables 1 and 2 summarize the studies that use lipid-based nanocarriers to deliver immunotherapy-based drugs, including Table 2 those studies focused on hematological cancers.

**Table 1:** Examples of lipid-based nanosystems used to deliver immunotherapies

**Lipid-based nanosystem**	**Cancer treated**	**Therapeutic delivered**	**Main results**	**Ref**
DOTAP/cholesterol (CHOL)/DOPE-based cationic liposomes	Breast Cancer	AE36 peptide and CpG ODN adjuvant (cancer vaccine)	CD8^+^/CD4^+^ immune responses, IL-4/IFN-γ cytokine secretion, and significant tumor reduction with an increased survival time	[18]
DSPC/DSPG/DSPE/DOPE/CHOL-based liposomes	Breast Cancer	AE36, E75, E75-EA36 and PADRE (adjuvant) peptides (cancer vaccine)	CD8^+^/CD4^+^ immune responses, IFN-γ cytokine secretion, tumor growth inhibition and increased life expectancy	[19]
Cationic pH-sensitive liposome (DC-CHOL/DOPE/PEG-phosphatidyl-ethanolamine/phosphatidyl-choline (PC)/CHOL)	Melanoma	OVA antigen and nucleic acid-based adjuvants (CpG ODN and cGAMP) (cancer vaccine)	Release at intracellular acidic pH. Type I/II interferon and Th1-type cytokine secretion and significant tumor regression	[21]
PEGylated liposomes (hydrogenated soy phosphatidylcholine (HSPC)/mPEG2000-DSPE/CHOL)	Colon carcinoma	Checkpoint inhibitor (anti CTLA-4)	Tumor accumulation. Increased survival time, tumor growth delay, higher Teff/Tref ratio, and increased CD8^+^ T cell number	[22]
HSPC/CHOL/DSPE-PEG2000-based immunoliposomes (first time as immunotherapy)	Melanoma	Checkpoint inhibitor (anti PD-L1 Fab)	Tumor targeting and accumulation. Secretion of CD8^+^ T cells. Total regression (20%) and tumor growth delay (40%)	[23]
pH-sensitive DSPE-based immunoliposomes	Melanoma	Checkpoint inhibitor (anti PD-L1) and catalase	Tumor targeting and accumulation. Reduced tumor hypoxia, increased CD8^+^ T cell number, tumor growth inhibition, and increased survival time	[24]
Exosomes from bone marrow-derived dendritic cells (DEXs)	Melanoma	OVA antigen and anti-CTLA-4	The carrier allowed the synergistic action of both immunotherapeutics. Significant reduction of tumor growth	[25]
Tumor-derived exosomes (TEXs) from engineering modified K562 tumor cells	-	-	Can express IL-15, IL-18 and 4-1BBL proteins on their surface. Activation of NK cells in a short-time window	[27]

**Table 2:** Examples of lipid-based nanosystems used to deliver immunotherapies. Case study: hematological cancers

**Lipid-based nanosystem**	**Cancer treated**	**Therapeutic delivered**	**Main results**	**Ref**
Ionizable lipid nanoparticles (LNP)	Leukemia	mRNA capable of inducing temporary CAR expression in T cells	Equivalent expression compared to electroporated CAR T cells with lower toxicity. Capacity to kill cancer cells	[28, 29]
Charge-altering releasable transporters (composed by lipid blocks and a charge-altering block)		mRNA	Effective transfection of NK cells. mRNA release at pH 7.4. Without off-target effects. Generation of cytotoxic anti-CD19 CAR NK cells	[30]
DOTAP-based cationic liposomes		Anti-IL-10 siRNA and tumor antigens (cancer vaccine)	DC maturation/IL10 inhibition. Strong cytotoxicity. Cell burden and metastasis reduction. Increased survival	[31]
CHOL/1,2-dipalmitoyl-sn- glycero-3-PC (DPPC)/DSPE-PEG2000-based liposomes		Anti-CD3 and anti-CD33 (BiTE)	Alternative to BiTEs/CAR T cells. Targeting and cytotoxic capacity. Tumor load reduction and increased survival	[32]
Genetically modified exosomes (TEXs)		-	PD-L1 inhibition. Significant T cell activation and proliferation with potent cytotoxic CD8^+^ response. Reduced tumor growth and extended survival	[33]
Exosomes (TEXs)		-	Modification of gene expression of CD8^+^, CD4^+^ and CD4^+^CD39^+^ T cells. Significant CD8^+^ immune responses	[34, 35]
CHOL/L-α-phosphatidylcholine (PC)-based liposomal formulation	Lymphoma	OVA/adjuvants (poly(I:C) and CpG ODN) (cancer vaccine)	Synergistic effect of the adjuvants with the carrier. Immunization for 8 months with deceleration in tumor progression	[20]
C3-targeted liposomes (DPPC/1,2-distearoyl-sn-glycero-3-phosphocholine (DSPC)/DSPE-PEG2000)		OVA antigen (cancer vaccine)	Antigens delivered and internalized in APCs. Activation of significant number of T cells. Reduced tumor growth and potent antitumor immune responses	[36]
DOPE/DSPE-PEG1000/CHOL derivative-based small LNP		OVA derivative and CpG (cancer vaccine) and anti-PD1 checkpoint inhibitor	Strong antitumor activity. Avoidance of the reappearance of cancer cells due to action of both immunotherapeutics	[37]
PEGylated G0-C14-based LNP		Synthetic mRNA that encodes OVA and R848 adjuvant (cancer vaccine)	Transfection efficiency: ≥ 95%. Potent MHC class-I antigen presentation. Significant adaptive immune response and tumor growth reduction	[38]
LNP (ionizable cationic lipid/PC/CHOL/PEG lipid)		mRNA that encodes anti-CD20	In situ antibody expression. Tumor growth reduction and enhanced survival	[39]
Phospholipid-based nanoparticles		OX40/CD137 mRNA and anti-OX40	Complete response rate of 60%. No regression with re-exposition	[40]
CHOL/DPPC/DSPE-PEG2000-based liposomes	Multiple myeloma	Anti-CD3 and BCMA, CS1 and/or CD38 (BiTEs)	Alternative to CAR technology. Increase half-life of BiTEs	[43]
DPPC/CHOL/PEG2000-PE-based liposomes		Phytohemagglutinin	High T cell activation. Reduced tumor progression and enhanced survival	[44]

Still, to successfully achieve the translation of the liposomal formulations of immunotherapeutics to the clinical practice some challenges need to be addressed, such as increasing their low loading capacity, solving their stability problems, including the oxidation of phospholipids, and controling the burst release processes, among others.

**Fig. 1:**
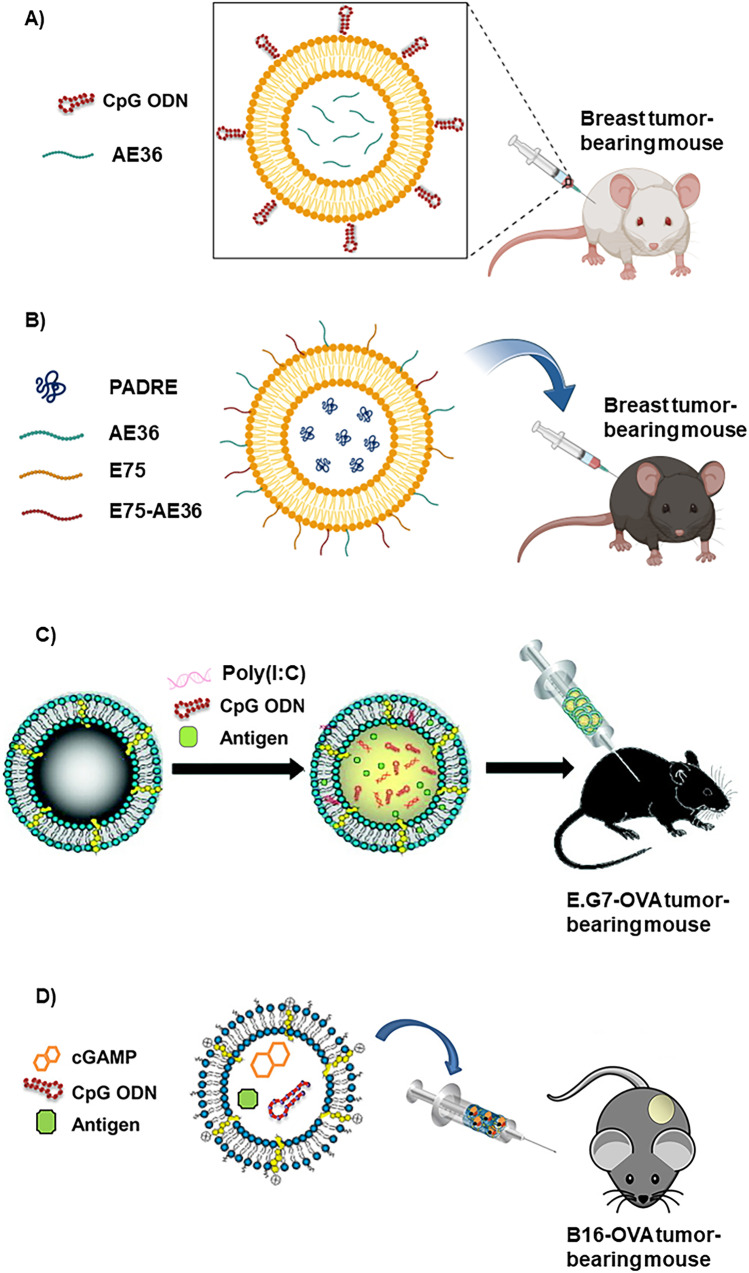
Examples of the capability of liposomes to accommodate all components of a therapeutic vaccine. **A** Peptide-based vaccine formulated as a cationic liposomal formulation composed of DOTAP, cholesterol, and DOPE that includes a HER2/neu-derived peptide (AE36) inside and an adjuvant (CpG-ODN) in the surface [18]. Created with BioRender.com. **B** Liposomal peptide-based cancer vaccine composed of DSPC, DSPG, DSPE, DOPE, and cholesterol that includes the AE36, E75, and E75-AE36 peptides in the surface and the PADRE peptide inside [19]. Created with BioRender.com. **C** Liposomal vaccine for the codelivery of two nucleic acid-based adjuvants, CpG ODN and Poly(I:C), which target TLR9 and TLR3, and the OVA antigen. Reprinted from reference [20] with modifications with permission. **D** Liposomal pH-sensitive vaccine for the codelivery of two nucleic acid-based adjuvants, CpG ODN and cGAMP, which target TLR9 and STING. Reprinted with modifications from reference [21] with permission

The capacity of liposomes to incorporate hydrophilic molecules, such as proteins, peptides, and nucleic acids, has been used to facilitate the delivery of cancer vaccines to lymph nodes and the spleen. Once reaching their target, they promote their internalization into the cytosol of dendritic cells and induce the antigen cross-presentation process [17]. Several examples illustrating the ability of liposomes to accommodate all components of a therapeutic vaccine can be seen in Fig. 1 and are described below in the text.

Regarding peptide-based cancer vaccines, Barati et al. incorporated a human epidermal growth factor receptor 2 (HER2/neu)- derived peptide (AE36) and an adjuvant, cytosine-phosphate-guanine (CpG), in a cationic liposomal formulation composed of N-[1-(2,3-dioleoyloxy)propyl]-N,N,N-trimethylammonium methyl-sulfate (DOTAP), cholesterol, and 1,2-dioleoyl-sn-glycero-3-phosphaethanolamine (DOPE) (Fig. 1A). This peptide-based cancer vaccine was evaluated in a HER2^+^ breast cancer mouse model, resulting in a promising immune response of CD8^+^ and CD4^+^, a significant cytokine secretion of IL-4 and IFN-γ, and a notable reduction in tumor size and increase of survival time [18]. Similarly, Zamani et al. reported a liposomal peptide-based cancer vaccine that simultaneously delivered HER2/neu-derived peptides (AE36 and E75), a multi-epitope peptide (E75-AE36), and a Pan HLA DR-binding epitope (PADRE) peptide. The authors proposed a formulation composed of DSPC (1,2-distearoyl-sn-glycero-3-phosphocholine), DSPG (1,2-distearoyl-sn-glycero-3-phosphoglycerol), DSPE (1,2-distearoyl-sn-glycero-3-phosphoethanolamine), DOPE, and cholesterol, which allowed the conjugation of AE36, E75, and E75-AE36 peptides in the surface of the nanocarrier and the encapsulation of the PADRE peptide, which behaves as an effective adjuvant, inside the nanocarrier (Fig. 1B). This cancer nanovaccine was subcutaneously injected on HER2^+^ breast tumor-bearing mice and generated CD4^+^ and CD8^+^ immune responses, IFN-γ secretion, and a significant decrease of tumor growth and increase of life expectancy. Notably, E75-AE36 and PADRE peptides were only significantly effective in increasing the antitumor immune responses when administered in the liposomal form, which suggests that this type of carrier acts as a valuable adjuvant [19].

Liposomes can be used to formulate vaccines that include multiple nucleic acid-based adjuvants against different Toll-like receptors (TLRs) and STING (stimulator of interferon genes) receptors. For example, Bayyurt et al. reported the co-delivery of ovalbumin (OVA) antigen with two nucleic acid-based adjuvants, CpG oligodeoxynucleotide (CpG ODN), which targets TLR9, and poly(I:C) (polyinosinic-polycytidylic acid) that targets TLR3, by means of a liposomal formulation (Fig. 1C). The synergistic effect of the adjuvants and the liposome structure facilitates the carrier internalization and the activation and maturation of dendritic cells, resulting in a specific immune response that generated, for at least eight months, the immunization of E.G7-OVA tumor-bearing mice, ultimately showing a significant reduction in tumor progression [20].

A very similar example is the cancer vaccine (Fig. 1D) that was formulated by a pH-sensitive liposome and includes, apart from the model antigen, OVA, two nucleic acid-based adjuvants, CpG ODN and cGAMP (2′,3′-ciclic-guanosine monophosphate-adenosine monophosphate) that act synergistically, using two different pathways against TRL9 and STING receptors, respectively. The cationic pH-sensitive nanocarrier permitted the delivery of their cargo towards the antigen-presenting cells and its internalization, being released after the destabilization of liposomes at the intracellular acidic pH, resulting in an important immune response with a significant secretion of type I and II interferon and Th1-type cytokines from the antigen-presenting cells and an important tumor regression in B16-OVA tumor-bearing mice [21].

The inclusion of checkpoint inhibitors inside liposomes can improve their tumor accumulation and efficacy, avoiding the multiple immune-related adverse events that have been attributed in literature to this immunotherapy approach. In this regard, Nikpoor et al. evaluated anti-CTLA-4 (cytotoxic T-lymphocyte antigen-4) antibody-loaded PEGylated liposomes in C26 colon tumor-bearing mice. They found that the encapsulation of this blocking antibody significantly increased their tumor accumulation throughout the enhanced permeability and retention (EPR) effect. Remarkably, the vesiculation of the CTLA-4 inhibitor in PEGylated liposomes led to a 5-day increase in the median survival time (MST), a tumor growth delay (TGD) of 11.8%, a higher effector T cells (Teff) to regulatory T cells (Treg) ratio and a greater CD8 + T cells number [22].

Similar approaches were reported to inhibit the PD-1/PD-L1 (Programmed Death 1/Programmed Death Ligand 1) pathway by modifying liposomes’ surface with different anti-PD-L1 and analogs to obtain immunoliposomes. For example, Merino et al. optimized the conjugation of anti-PD-L1-Fab (fragment antigen-binding) to the surface of liposomes, showing a high targeting capacity to B16OVA tumor cells and significant tumor accumulation in B16OVA tumor-bearing mice. This system was able to increase the secretion of effector CD8 + T cells that led to the shrinkage of the tumors and generated total regression and tumor growth delay in 20% and 40% of mice, respectively. Interestingly, this was the first time that immunoliposomes were reported as an immunotherapy approach and not just for targeting purposes [23]. Likewise, Hei et al. described a multifunctional immunoplatform to effectively inhibit the PD-1/PD-L1 pathway in B16F10 tumor-bearing mice. They attached an anti-PD-L1 antibody to the surface of DSPE-based liposomes and included catalase in the interior of the carrier to overcome tumor microenvironment hypoxia and thereby enhance the efficacy of the checkpoint inhibitor. The formulation provided protection to the enzyme and targeting capacity to the antibody, resulting in an enhanced anti-PD-1 tumor accumulation and a reduced tumor hypoxia with an increased number of CD8 + T cells; additionally, there was a reduced tumor growth and an increased survival time in vivo [24].

The recent use of extracellular vesicles, mainly exosomes, as a strategy to enhance the main immunotherapy approaches, is due to their unique natural origin as they are obtained directly from living cells, showing low toxicity and non-immunogenicity when utilized. In this regard, since dendritic cell-derived exosomes (DEXs) have presented promising immune responses, they can be used as cancer nanovaccines, although they must overcome the immunosuppressive micro-environment of tumors to reach the clinical practice. Phung et al. reported exosomes generated from bone marrow-derived dendritic cells pulsed with OVA and functionalized with the checkpoint inhibitor anti-CTLA-4. The synergistic action of both immunotherapy-based strategies causes the formulation to reach the lymph nodes and be bond specifically with T cells, resulting in a significant reduction of tumor growth in B16-OVA tumor-bearing mice [25]. Since DEXs are generated from dendritic cells pulsed with tumor antigens, they can express, among others, MHC-I and II molecules and co-stimulatory molecules, such as CD40, CD80, and CD86, being able to be used with the same functionality of the original dendritic cells. For that, clearly, DEXs are the alternative to the tumor-derived exosomes (TEXs) that contain, in addition to tumor neoantigens that can generate important immune responses, molecules with the capacity of promoting immune escape, and cancer progression [26]. Nevertheless, in recent years, numerous efforts are being made to strengthen the immuno-stimulatory properties of TEXs throughout different engineering approaches. For example, Li et al. described TEXs generated from engineering-modified K562 tumor cells. These exosomes were able to co-express the 4-1BB ligand (or TNFSF9, tumor necrosis factor ligand superfamily member 9) and the IL-15 and IL-18 molecules, which activate the NK cells that increase their activity and proliferation in a short-time window (4 h). However, after 48 h of treatment, the opposite effect was produced [27].

#### Addressing immunotherapy for hematological cancers with lipid-based carriers

Despite the many advantages described above in the text of using lipid-based nanocarriers for the improvement of the immunotherapy approaches in the treatment of cancer, there are only a few studies that have explored this for hematological cancers, which are collected in Table 2.

In the case of leukemia, Billingsley et al. used ionizable lipid nanoparticles (iLNP) as a promising alternative to the current methods for the ex vivo modification of T cells by the CAR technology. They reported mRNA-loaded iLNP that were capable of inducing a temporary CAR expression in human T cells, equivalent to that achieved with electroporated CAR T cells, but with much lower toxicity, finding that both approaches had a strong ability to kill cancer cells in Nalm-6 acute lymphoblastic leukemia (ALL) cells [28, 29]. Similarly, Wilk et al. used mRNA-loaded lipid-based nanoparticles to achieve the effective transfection of natural killer (NK) cells transiently without its activation or reconfiguration. The nanoparticles were composed of several lipid blocks and a “charge-altering block,” which permit the complexation of mRNA into the nanoparticles and its release at physiological pH. This formulation avoided the off-target effects of electroporation techniques and resulted in the generation of anti-CD19 CAR NK-cells in vitro that were highly cytotoxic in CD19 + Nalm-6 cells [30].

Additionally, Iversen and Sioud reported the ex vivo delivery of anti-IL-10 siRNA and tumor antigens to immature dendritic cells (DCs) by means of DOTAP-based cationic liposomes to enable the DCs maturation and inhibit IL-10 expression. This cancer vaccine triggered the killing of leukemia cells, decreased cell burden and metastasis, and increased survival in a Brown Norway rat model of acute myeloid leukemia (AML) [31]. Moreover, Alhallak et al. reported a new bispecific T cell engager (BiTE) generated by conjugating the T cell targeting molecule anti-CD3 and the acute myeloid leukemia targeting monoclonal antibody anti-CD33 to the surface of a liposome as an alternative to conventional BiTEs and CAR T cells. This nanoplatform specifically targeted the acute myeloid leukemia cells in vitro and induced its death in vivo by the activation of T cells, reducing the tumor load of mice and increasing their survival time [32]. Besides, Huang et al. described the optimization of leukemia cell-derived exosomes, by silencing PD-L1 expression in the acute lymphocytic leukemia cells. The genetically modified TEXs induced T cell activation and proliferation with a potent cytotoxic CD8 + response. Vaccination of L1210 tumor-bearing mice with this nanosystem extended the survival of the animals and reduced tumor growth [33]. In this regard, Pando et al. have recently reported the modification of gene expression of CD8 + , CD4 + , and CD4 + CD39 + T cells by using AML-derived extracellular vesicles. The authors reported in vitro and in vivo the impact of these TEXs on the induction of significant CD8 + immune responses [34, 35].

In the case of lymphoma, apart from the above-described study of Bayyurt et al. [20], only five other studies have been conducted using lipid-based nanocarriers in the last 5 years to the best of our knowledge. Francian et al. reported a liposomal formulation that facilitates the delivery of antigens to antigen-presenting cells (APCs) by including the pyridyldithiol propionate group that reacts with the sulfhydryl group of the complement C3 proteins in serum that binds to the C3 receptors of APCs. Thus, C3 receptor-targeted liposomes loaded with OVA were internalized in APCs, generating the activation of a significant number of T cells. The administration of this formulation in A20-OVA tumor-bearing mice resulted in a reduced tumor growth and in a potent antitumor immune response [36]. Also, Kim et al. developed a nanovaccine based on small lipid nanoparticles (SLNPs) that produced strong anti-tumor activity in E.G7-OVA tumor-bearing mice. This SLNPs-based vaccine was formulated using two phospholipids, DOPE to facilitate endosomal escape and DSPE-PEG_1000_ to enhance the stability of the carriers and fix the model antigen (a derivative of OVA) to them, and a cholesterol derivative, monoarginine-cholesterol, to facilitate the adjuvant (CpG) complexation with the nanocarrier. Although the vaccine produced an increased expression of PD-L1 that reactivates the tumor, the sequential administration of the nanovaccine and their combination with an anti-PD-1 checkpoint inhibitor suppressed the reappearance of cancer cells in the animals [37]. Besides, Islam et al. reported PEGylated G0-C14-based lipid nanoparticles for the co-delivery of a synthetic mRNA that encodes the OVA antigen and the R848 adjuvant that targets TLR7/8. This mRNA-based nanovaccine achieved a transfection efficiency of more than 95% and a potent MHC class-I antigen presentation in vitro in APCs, and an important adaptive immune response in vivo, resulting in a substantial reduction of tumor growth in an EG.7-OVA lymphoma animal model [38].

Likewise, Thran et al. described the use of mRNA-loaded LNP that encodes the anti-CD20 monoclonal antibody to achieve an in situ antibody expression in a Burkitt’s lymphoma animal model. After the repeated administration of this system, tumor growth was significantly reduced and almost all animals survived after 28 days [39]. Other authors focused on transferring T cell co-stimulatory receptor (OX40/CD137) mRNA to tumor-infiltrating T cells by phospholipid-based nanoparticles (PLNPs) as these receptors are often downregulated in these immune cells, limiting the use of antibodies as an immunotherapeutic strategy. The combined use of the OX40-mRNA-loaded PLNPs and anti-OX40 antibodies resulted in a complete response rate of 60% in an A20 tumor model. Furthermore, when the mice were re-exposed to tumor cells, they showed no regression [40]. In this regard, currently, there are two ongoing phase I clinical trials about the use of mRNA-loaded LNPs that encode OX40L (mRNA-2416, NCT03323398) or OX40L/IL-23/IL-36γ (mRNA-2752, NCT03739931), alone or in combination with the anti-PD-L1 durvalumab, for the treatment of lymphoma [41, 42].

Finally, with respect to multiple myeloma, only two recent studies by Alhallak et al. were found. In the first article, the authors used liposomes to generate three nanocarrier-based bispecific T cell engagers (BiTEs) as an alternative to the CAR technology. The carrier was attached to the T cell targeting molecule, anti-CD3, and to different monoclonal antibodies that target a multiple myeloma antigen (BCMA, CS1, or CD38), increasing the poor half-life of conventional BiTEs. Moreover, these authors synthesized a new carrier-based entity with the same functions as the conventional BiTEs but capable of targeting multiple antigens of multiple myeloma at once by the functionalization of the surface of liposomes with one monoclonal antibody that is directed to CD3 and other three that target the antigens BCMA, CS1, and CD38. Both liposome-based entities had a sufficiently long lifetime to allow the maintenance of the therapeutic activity with a weekly administration, although the multi-targeting capacity of the latest formulation was more effective in multiple myeloma in vitro and in vivo than the first one [43]. Moreover, the same authors reported a promising strategy to overcome the poor T cell activation of the T cell engagers by using phytohemagglutinin (PHA). When including this lectin in liposomes, their instability and toxicity were decreased. Liposomal PHA generated high values of T cell activation with a significantly reduced tumor progression and prolonged survival time in a multiple myeloma animal model [44]. Nevertheless, two other studies explored the possibility of acting on NK cells by multiple myeloma-derived exosomes that may be released by the action of genotoxic agents, like melphalan [45, 46].

### Use of polymer-based nanocarriers in immunotherapy treatment

#### Overview

Polymeric nanoparticles can be synthesized from natural, synthetic, or semi-synthetic materials. This variety of materials, which usually are very well accepted and tolerated by the organism, offers a wide range of options for transporting multiple immunotherapeutics. Tables 3 and 4 include some examples that use polymer-based nanocarriers to deliver immunotherapy-based drugs, Table 4 being focused on those studies related to hematological cancers. Compared to other nano-scale structures, these carriers have substantially higher loading capacities, are more stable, and permit better control of their physicochemical properties and over the release of their payloads by means of different mechanisms, such as diffusion, degradation, or erosion [47].

**Table 3:** Examples of polymer-based nanosystems used to deliver immunotherapies

**Polymer-based nanosystem**	**Cancer treated**	**Therapeutic delivered**	**Main results**	**Ref**
PEI-dextran-based nanoparticles	Breast cancer	-	Polymers modulated myeloid-derived suppressor cells towards an antitumor phenotype with restoration of T cell activity and tumor growth reduction	[51]
PEI-based nanocarrier	Melanoma	OVA and CpG adjuvant (cancer vaccine)	PEI produced antigen uptake by DCs and endosomal escape. Enhanced CD8^+^ T cells expression	[52]
Glycol chitosan and PLGA or PEG-PLGA-based nanocarriers	Melanoma	OVA and adjuvants (MPLA and CpG) (cancer vaccine)	Controlled antigen release in cytosol. Efficient uptake by DCs. Robust T cell activation and immune responses	[53]
Polymer-based ultra-pH-sensitive nanoparticles	Melanoma, colon cancer and papilloma virus tumor	OVA antigen	Antigen delivery to DCs cytosol. The nanocarriers acted as adjuvants. Tumor growth inhibition. Synergistic effects with anti-PD-1 (lung cancer)	[58]
PEG-PLGA-based nanoparticles	Colorectal cancer	Anti-PDL1 (checkpoint inhibitor)	Enhanced tumor targeting and half-life of anti-PDL1. Tumor growth inhibition and significant immune response	[59]
Methoxy-PEG-PLGA nanoparticles	Melanoma	Anti-PDL1 (checkpoint inhibitor)	Significant antitumor immunity. Reduced dose of checkpoint inhibitor	[60]
Poly(β-L-malic acid)-methoxy-PEG5000-based targeted nanoparticles	Glioblastoma	Ipilimumab and nivolumab	Checkpoint inhibitors can cross BBB due to the targeting ligand of carriers (transferring R). Increased number of CD8^+^ T cells and NK cells in the brain	[61]

**Table 4:** Examples of polymer-based nanosystems used to deliver immunotherapies. Case study: hematological cancers

**Polymer-based nanosystem**	**Cancer treated**	**Therapeutic delivered**	**Main results**	**Ref**
Targeted (antiCD3 fab)-PBAE-based nanoparticles (PBAE was modified with a peptide to facilitate transfection)	Leukemia	Plasmid DNA encoding leukemia-specific CAR genes	Delivery of therapeutics to T cells nuclei to efficiently reprogram them. Alternative to ex vivo CAR technology with similar tumor regression efficacies	[62]
		mRNA	T cell genetic modification to produce CD19-targeted 41BBζ CAR T cells	[63]
PBAE-based nanoparticles		In vitro-transcribed CAR mRNA	In situ T cell temporary reprogramming Similar tumor regression efficacies that ex vivo CAR technology	[64]
Methoxy-PEG/poly(2-ethylbutyl phospholane/PEI-based micelles		Anti-CD19 CAR-expressing plasmid	Transfection efficiency similar to Lipofectamine 2000™ with a reduced toxicity. Immune-mediated cytotoxicity	[65]
AML-coated PLGA-based nanoparticles		AML-specific antigens and CpG-derivative adjuvant	Increased specific immune responses. Prophylactic protection. Adjuvant treatment after chemotherapy	[66]
Polyanhydrides-based nanoparticles	Lymphoma	OVA and PELA adjuvant (cancer vaccine)	Polymer/PELA synergistic effect that produced additive immune responses. Prophylactic effects with CD8^+^ T cells expression and tumor growth reduction	[48]
γ-PGA-based nanoparticles		OVA and poly(I:C) adjuvant (cancer vaccine)	Immune response with significant activation of NK cells and CD8^+^ T cells and tumor growth inhibition	[50]
PLGA-based nanoparticles	Lung cancer and lymphoma	Peptide-based vaccines in combination with poly(I:C)/resiquimod—MIP3α-loaded particles	The sustained release of the adjuvants that acted as immunomodulators resulted in an increased efficacy of two peptide vaccines	[54]
PEOz-PLA/carboxyl-terminated-Pluronic F127-based micelles	Lymphoma	OVA and CL264 adjuvant (cancer vaccine)	Scavenger receptor-mediated endocytosis. Endosomal escape. High immune responses and immunization	[56]
		Peptide model antigen	Activation of the complement system. Antitumor immunity. Tumor growth reduction and enhanced survival	[57]
PLGA or chitosan-based nanoparticles		OVA and poly(I:C) adjuvant (cancer vaccine)	Increased number of activated OVA-specific CD8^+^ T cells. Significant anti-tumor efficacy and prolonged survival	[67, 68]
PEG-PGA-based nanoparticles		OVA and IL-7	Antigen-specific antitumor immune responses with tumor regression. Significant prophylactic effect with reduced growth of tumor cells	[69]
Poly(propylene sulfide)-based nanoparticles		OVA and CpG adjuvant (cancer vaccine)	Potent CD8^+^-based immune responses, significant tumor regression and enhanced survival	[70]
Polyethylenimine-based polymer conjugate	Lymphoma	Rituximab and tositumomab	Binding affinity with reduced off-target effects and minimal bold clearance. Long-term survival with significant tumor growth inhibition	[71]
Targeted CXCL13-zwitterionic particles (2-methacryloyloxy-ethyl phosphorylcholine monomers and glycerol dimethacrylate crosslinkers)		Rituximab	Ability to cross BBB and reach the CNS. pH-responsive sustained release. Ten-fold accumulation of the antibody compared to free drug. Control of CNS metastases and tumor removal	[72]
PEI and polypyrrole-based nanocomplex		Drug-free	Lysosomal membrane permeabilization, loss of mitochondrial transmembrane potential and apoptosis. Long-term tumor growth inhibition	[74]
Poly(phosphorhydrazone) dendrimers functionalized with amino-bis(methylene phosphonate)	Multiple Myeloma	-	Ex vivo NK cell proliferation and expansion. Significant cytotoxic effects	[75]
PLGA-based nanoparticles		BCMA-based peptide (peptide-based vaccine)	Activation of BCMA-specific CD8^+^ T cells. Increased antitumor responses	[76]
PEGylated rhIL-15 (NKTR-255 polymer conjugate)	Lymphoma and Multiple Myeloma	IL-15	Enhanced pharmacokinetics. Potent immune responses with significant antitumor effects and increased survivals. Clinical trials: NCT04136756 and NCT05359211	[77, 78]

Biodegradable and biocompatible polymers have been used in carrier-based cancer vaccines for the co-delivery of antigens and adjuvants to dendritic cells, behaving themselves as an adjuvant. In this regard, Wafa et al. demonstrated the synergistic effect of polyanhydrides and the adjuvant pentaerythritol lipid A (or PELA), which is a safer derivative of the TLR4 agonist monophosphoryl lipid A (MPLA), to deliver the model antigen ovalbumin (OVA) to dendritic cells, activate them, and produce additive immune responses, since both are able to bind to TLR4 of these APCs. This formulation showed prophylactic effects in E.G7-OVA tumor-bearing mice that displayed an increased expression of antigen-specific CD8 + T cells with a decrease in the tumor growth rate [48].

Other biodegradable polymers, like chitosan or poly(γ-glutamic acid) (γ-PGA), can help modulate immature dendritic cells and macrophages towards an immunostimulatory activity with an increased proliferation of T cells that decreases cell invasion profiles, without affecting the viability or the metabolic activity of the APCs. These results make both polymers attractive materials for carrier-based vaccines by combining their capacity to incorporate and deliver antigens and adjuvants and to act by themselves as adjuvants [49]. By modifying the carboxyl functional groups of γ-PGA-based nano-scale particles with amine moieties, Kim et al. managed to deliver the OVA model antigen and the adjuvant poly(I:C) to the lymph nodes in E.G7-OVA tumor-bearing mice, resulting in an important immune response, with a significant activation of NK-cells and CD8^+^ T cells, and a subsequent tumor growth inhibition [50].

Some cationic polymers, such as polyethyleneimine (PEI) and cationic dextran, have shown their capacity to modulate by TLR4 signaling the immunosuppressive profile of myeloid-derived suppressor cells (MDSCs) towards an anti-tumor phenotype with an effective restoration of T cell activity and a reduction of the tumor growth in 4T1-tumor-bearing mice [51]. Likewise, Guan et al. formulated a simple nanovaccine by the electrostatic interaction of PEI with the antigen OVA and the adjuvant CpG, being the cationic polymer responsible for the antigen uptake by the dendritic cells and for the antigen endosomal escape due to the “proton sponge” effect that facilitates antigen cross-presentation and enhances the expression of antigen-specific CD8 + T cells [52].

Thus, the use of polymers, such as polyanhydrides, chitosan, PGA, PEI, or dextran, as materials for nanovaccine preparation, enhances the common cancer vaccines by acting themselves as adjuvants in synergy with those that accompany the antigens, activating humoral and cellular immunogenicity and modulating the immune profiles.

Nanoparticulate polymeric carriers can offer a sustained and controlled antigen release that increases their exposure to dendritic cells, facilitating the activation of these APCs. In this regard, Zupančič et al. synthesized polymeric nanoparticles based on glycol chitosan and poly(lactic-co-glycolic acid) (PLGA) or PEG-PLGA that incorporated two adjuvants, MPLA and CpG, and the model antigen OVA, which was encapsulated in the particles or adsorbed in their surface, with glycol chitosan being the component that increased the antigen loading. Both formulations were efficiently uptaken by dendritic cells through different pathways and antigen cross-presentation was facilitated by the functional groups of both polymers that generated the release of the antigen in the cytosol by the “proton sponge effect.” The formulation produced a robust T cell activation with important immune responses in mice bearing B16.M05 tumor cells because of the coordinated release of the adjuvants and the antigen [53]. The effects of therapeutic cancer vaccines can be enhanced by the sustained release of multiple adjuvants that act as immunomodulators from polymer-based nanocarriers. In fact, Silva et al. reported the increased efficacy of two peptide-based vaccines in lung and lymphoma tumor models when used in combination with PLGA-based nanoparticles loaded with two TLR agonists, poly(I:C) and resiquimod, and the chemokine MIP3α (macrophage inflammatory protein-3 alpha) [54].

**Fig. 2:**
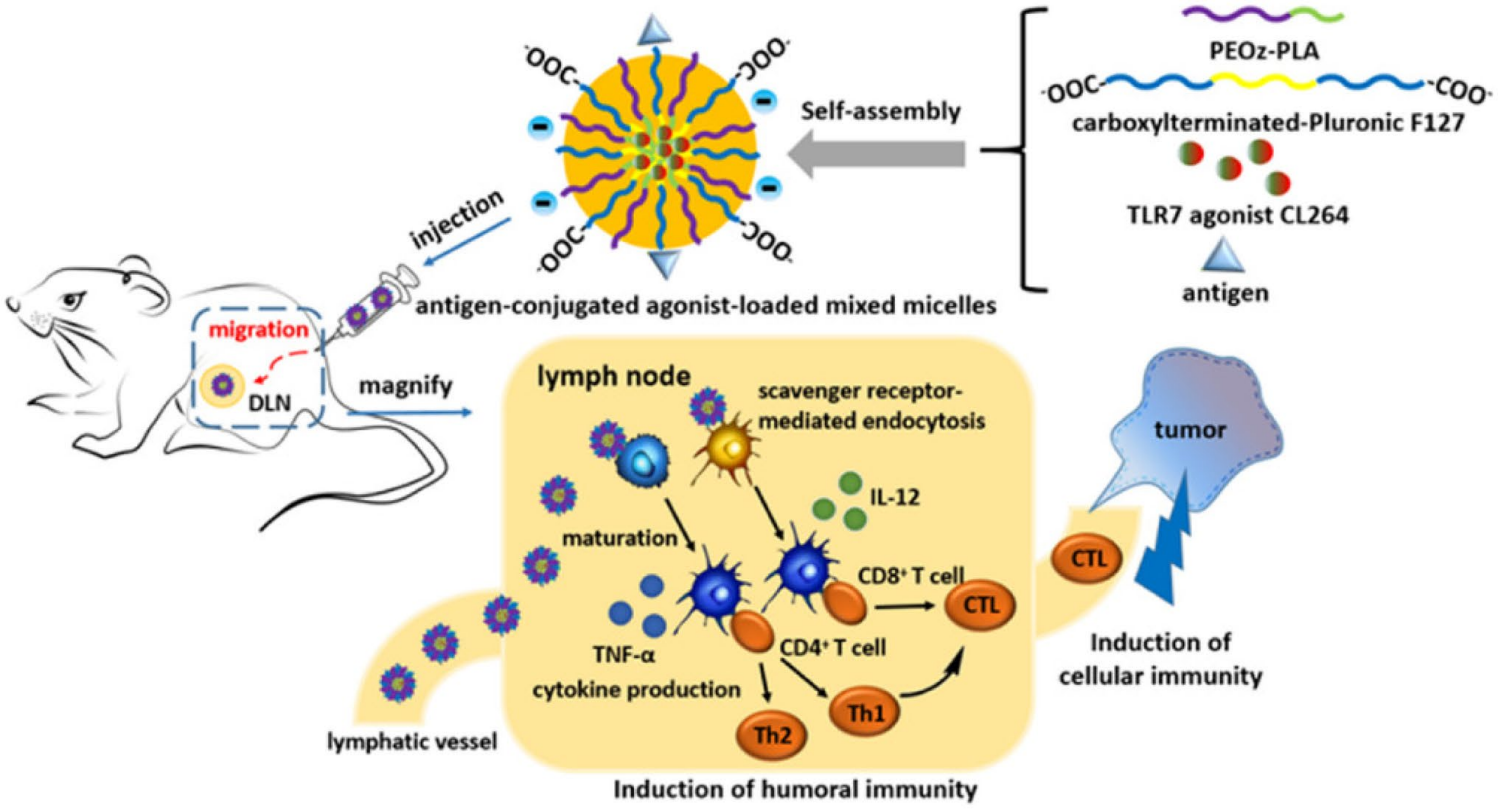
Scheme of the preparation of the OVA/CL264-loaded pH-sensitive micelles reported by Li et al. and hypothesis of the mechanism of the codelivery of the OVA antigen and the TLR-7 agonist CL264 to the dendritic cells in the lymph nodes and their internalization in dendritic cells by scavenger receptor-mediated endocytosis. Reprinted from reference [56] with permission

Furthermore, the hydrophobicity of some polymers, such as poly(lactic acid) (PLA) or PLGA, may facilitate the delivery of antigen-loaded carriers towards APCs, enhancing the immune response by an increased antigen uptake [55]. In this regard, Li et al. synthesized pH-sensitive micelles using poly(2-ethyl-2-oxazoline)-PLA (PEOz-PLA) and carboxyl-terminated-Pluronic F127 to achieve the codelivery of the OVA antigen and the TLR-7 agonist CL264 to the dendritic cells in the lymph nodes, with the carboxylic groups of the polymeric micelles being responsible for their internalization in dendritic cells by scavenger receptor-mediated endocytosis as shown in Fig. 2. Because of the adjuvant and the optimized composition of the nanomicelles, the formulation showed potent immune responses in vivo, causing the PEOz group an enhanced antigen presentation and the pH-sensitive PEOz-PLA the endosomal escape of the antigen. This system demonstrated a significant immunization ability in E.G7.OVA tumor-bearing mice [56]. The same authors demonstrated in a subsequent publication that a similar formulation was capable of delivering a peptide model antigen to dendritic cells in lymph nodes without using an adjuvant since the external hydroxyl groups of the micelles activated the complement system that produces antitumor immunity, acting the formulation as an adjuvant. The system reduced tumor growth and enhanced survival in E.G7-OVA tumor-bearing mice [57]. Antigen delivery to the cytosol of dendritic cells in lymph nodes has been achieved by means of polymer-based ultra-pH-sensitive nanoparticles. The transition pH of the copolymer used and its structure make the nanoparticles an adjuvant to the antigen with a comparable or better effectiveness than the conventional ones, causing its effect to be dependent on STING and not on the TLRs. Using these polymer-based nanovaccines, it was reported substantial tumor growth inhibition in various tumor models and important synergistic effects when used in combination with checkpoint inhibitors [58].

Like lipid-based nanocarriers, polymeric nanoparticles can increase the small proportion of the administered monoclonal antibodies that target the tumoral tissue to accomplish the checkpoint inhibitor therapies, reducing the off-target toxicities and the immune-related adverse events and increasing the half-life of these immunotherapeutic drugs. For example, Lee et al. described the encapsulation of anti-PDL1 monoclonal antibodies (mAb) in PEG-PLGA-based nanoparticles. This formulation enhanced the tumor cell targeting and the circulation time of the checkpoint inhibitor and inhibited tumor growth in MC38 colorectal tumor-bearing mice, showing a significant immune response [59]. Similarly, Ordikhani et al. encapsulated anti-PD1 mAb in methoxy-PEG-PLGA nanoparticles to achieve its delivery and internalization in the dendritic cells of the spleen. The administration of this formulation in B16F10 tumor-bearing mice produced a significant antitumor immunity, being possible to significantly reduce the dose of the checkpoint inhibitor because of the carrier [60]. Moreover, Galstyan et al. succeeded in delivering ipilimumab and nivolumab to the brain by targeted polymeric conjugates based on poly(β-L-malic acid), methoxy-PEG5000, and the anti-mouse TfR (transferrin R) antibody, which help the nanocarrier to cross the BBB. Given that these checkpoint inhibitors are not capable of crossing the blood–brain barrier (BBB) by themselves, this formulation enables the treatment of intracranial GL261 glioblastoma. The authors reported a significant increase of CD8 + T cells and NK cells in the brain of GBM tumor-bearing mice and increased survival times if combinations of anti-CTLA-4 and anti-PD1 conjugates were used [61].

#### Dealing with hematological cancers using immunotherapy-loaded polymeric carriers

In the specific case of hematological cancers, only a few studies have been published on the application of polymer-based carriers in the improvement of immunotherapy-based therapies, which are collected in Table 4. The easy modification of the surface of the polymer-based carriers facilitates the targeted removal of hematological cancer cells, which are in circulation or located in the bone marrow, by using immunotherapeutics without provoking significant myelosuppression.

In particular, some authors have reported poly(β-amino ester) (PBAE)-based nanoparticles with the capacity to deliver leukemia-specific CAR genes to the nuclei of T cells as a promising alternative to the conventional ex vivo modification of T cells by the CAR technology. The polymer was modified with a peptide that includes microtubule-associated sequences and nuclear localization signals to facilitate the transfection of the CAR genes into the T cells. These authors achieved to efficiently reprogram circulating T cells, producing similar tumor regression efficacies that the conventional ex vivo CAR technologies in an animal model of B-cell acute lymphoblastic leukemia (ALL) [62]. Using the same PBAE-based formulation, these authors described the delivery of mRNA to T lymphocytes in order to produce their genetic modification and generate CD19-targeted 41BBζ CAR T cells [63]. The same group reported several years after the use of PBAE-based nanoparticles to deliver in vitro-transcribed CAR mRNA to T cells to temporarily reprogram them in situ against leukemia antigens. The repeated administration of the formulation in an ALL animal model resulted in similar tumor regression values compared to those obtained using the ex vivo CAR technology [64]. Similarly, Fan et al. synthesized polymeric micelles by using methoxy-PEG, poly(2-ethylbutyl phospholane (PEBP) and PEI to deliver an anti-CD19 CAR-expressing plasmid to Jurkat cells (immortalized T lymphocyte cells). The transfection efficiency was similar to Lipofectamine 2000™ but with a reduced toxicity due to the “proton sponge effect” of PEI. The CAR Jurkat cells obtained induced immune-mediated cytotoxicity on CD19-expressing K562 leukemia cells [65].

In the case of acute myeloid leukemia (AML), Johnson et al. reported PLGA-based nanoparticles that were coated with membrane material from AML cells as nanovaccines. This formulation showed the ability to co-deliver to APCs the AML-specific antigens of the membrane material included in the shell, together with a CpG-derivative adjuvant encapsulated in the PLGA-based core, resulting in increased specific immune responses to AML compared to control vaccination with leukemia cell lysates in vivo. This system showed prophylactic protection to AML and efficacy as adjuvant treatment after chemotherapy [66].

Regarding lymphoma, apart from the studies in E.G7-OVA and RMA T lymphoma tumor models described above [48, 50, 54, 56, 57], there are some references that report the use of polymer-based nanocarriers for immunotherapy. Han et al. used PLGA- and chitosan-based nanovaccines to deliver the model antigen OVA and the adjuvant poly(I:C) to the dendric cells and produce their activation and subsequent maturation. Both formulations produced a great quantity of activated OVA-specific CD8^+^ T cells, a significant anti-tumor efficacy and a prolonged survival in EG.7 tumor-bearing mice [67, 68]. Moreover, Toyota et al. combined the conjugation of the OVA model antigen on the surface of PEG-PGA-based nanoparticles with the inclusion of interleukin-7 inside them to generate antigen-specific antitumor immune responses with tumor regression in E.G7-OVA tumor-bearing mice. This formulation demonstrated a significant prophylactic effect with a reduced growth of E.G7-OVA tumor cells in vivo [69]. Besides, Jeanbart et al. demonstrated potent CD8 + -based immune responses, a significant tumor regression, and an enhanced survival in an E.G7-OVA animal model, targeting tumor-draining lymph nodes by poly(propylene sulfide)-based nanoparticles that contain the model antigen OVA and the CpG adjuvant in their surface. The small size of the nanoparticles permitted the intradermal delivery of this vaccine platform to the skin-draining lymph nodes [70].

With respect to the use of monoclonal antibodies in lymphoma, Li et al. developed a new polyethyleneimine-based polymer conjugate that included two different anti-CD20 monoclonal antibodies, rituximab and tositumomab. This nanoconjugate possesses the same binding affinity to non-Hodgkin lymphoma cells as the free antibodies but with reduced off-target effects and minimal blood clearance. The intravenous administration of this formulation in non-Hodgkin lymphoma-bearing mice results in a long-term survival with a significant inhibition of the tumor growth [71]. In the case of the central nervous system (CNS) lymphoma, Wen et al. encapsulated the monoclonal antibody rituximab in zwitterionic polymeric particles, which were synthesized using 2-methacryloyloxyethyl phosphorylcholine (MPC) monomers and glycerol dimethacrylate (GDMA) crosslinkers, to cross the blood–brain barrier and reach the CNS. The GDMA crosslinkers permit a pH-responsive sustained release of rituximab in the target site, resulting in a ten-fold accumulation of the antibody in the CNS compared to the free drug. The simple conjugation of CXCL13 to the surface of the particles directed them to CXCR5-expressing non-Hodgkin lymphoma cells, controlling the CNS metastases in a murine xenograft model of non-Hodgkin lymphoma and removing the CNS lymphomas in a humanized bone marrow-liver-thymus (BLT) animal model [72].

Additionally, Wang et al. reported a new approach to modify NK cells by glycoengineering. The new immune cells showed significant binding affinity and cytotoxicity to CD22 + lymphoma cells and reduced tumor growth in tumor-bearing mice. In order to achieve the engineered NK cells, the authors used the metabolic engineering and the glycol-polymer insertion with poly(acrylic acid) methods [73]. Also, very recently, a drug-free entity composed of PEI and polypyrrole (PPY) has been reported to be effective against B-cell lymphoma, inducing lysosomal membrane permeabilization, loss of mitochondrial transmembrane potential, and apoptosis. In vivo, this nanocomplex showed long-term tumor growth inhibition in Raji lymphoma-bearing mice [74].

Besides, only 2 studies have been reported for multiple myeloma to the best of our knowledge. Poupot et al. described the use of poly(phosphorhydrazone) dendrimers functionalized with amino-bis(methylene phosphonate) in ex vivo cultures of human peripheral blood mononuclear cells from healthy or multiple myeloma patients for NK cell proliferation. The authors proved the cytotoxic effects of these newly generated NK cells in vivo in K562-tumor-bearing mice. This protocol of NK cell expansion was presented as a promising alternative to the conventional ex vivo methods [75]. More recently, Bae et al. reported PLGA-based nanoparticles containing a specific BCMA-based peptide, which has a high affinity for binding to HLA-A2-positive multiple myeloma cells, with the intention of developing a peptide-based vaccine. The carrier facilitated the antigen delivery and presentation in dendritic cells and the subsequent activation of BCMA-specific CD8^+^ T cells, resulting in increased antitumor responses in multiple myeloma cell lines when compared to the free peptide [76].

Moreover, recently, the NKTR-255 polymer conjugate, a PEGylated version of the recombinant human interleukin-15 (rhIL-15), was developed. While NKTR-255 and the naked rhIL-15 showed similar in vitro properties, mainly affinity and signaling biology, the polymer conjugate presented an enhanced pharmacokinetic profile that led to potent immune responses with significant antitumor effects and increased survivals in lymphoma-bearing mice [77] and in a humanized multiple myeloma mouse model in combination with daratumumab [78], enhancing the effectiveness of CD19-targeted CAR-T therapies [79]. These promising results were the basis for the ongoing phase I clinical trial in relapsed/refractory non-Hodgkin lymphoma and multiple myeloma patients as monotherapy or in combination with rituximab or daratumumab (NCT04136756), and for the recently posted phase Ib clinical trial in relapsed or refractory large B-cell lymphoma in combination with CD19-targeted CART T cell immunotherapy (NCT05359211) [41].

### Hybrid-based nanocarriers applied in cancer immunotherapy

#### General observations

A strategy that is being followed lately to overcome the drawbacks of the previous systems and benefit from their advantages is the combination of materials, using hybrid-based nanocarriers. Some examples of the use of these systems to deliver immunotherapy-based drugs are included in Table 5. Despite the application of these materials in cancer immunotherapy is in its infancy, some exciting progress is being reported in the last years. However, clinical translation of hybrid-based nanocarriers will be still complicated until some issues, like their safety, toxicity, biodegradation, and metabolism, are solved, not to mention the problems for its large-scale production due to their complex composition.

**Table 5:** Examples of hybrid-based nanosystems used to deliver immunotherapies

**Polymer-based nanosystem**	**Cancer treated**	**Therapeutic delivered**	**Main results**	**Ref**
CD11c-targeted gold nano-cages/liposomes	Melanoma	Tyrosinase-related protein 2-derived antigen and MPLA adjuvant (cancer vaccine)	CD8^+^ T cells activation with generation of an immune response that inhibits tumor growth and metastasis. Facile in vivo tracking	[80]
Apolipoprotein E3-targeted PLGA/DMPC hybrid nanosystem		αOVA and imiquimod adjuvant (cancer vaccine)	Uptake into DCs by macropinocytosis. CD8^+^ T cells activation. Prophylactic effect in preventing metastasis	[83]
tLyp1 peptide-targeted PLGA/DSPE-PEG hybrid nanoparticles		Imatinib	Treg cells downregulation. Controlled drug release and increased tumor accumulation. Significant immune responses and antitumor effects when combined with anti-CTLA-4	[85]
Mannosylated carboxymethyl chitosan/protamine sulfate/calcium carbonate hybrid nanoparticles	-	CpG oligodeoxynucleotides	pH-sensitive intracellular release. Macrophages modulation towards M1 phenotype with antitumor activity. High levels of proinflammatory cytokines	[86]
RAW264.7 macrophages/4T1 cancer cells-coated PLGA nanoparticles	Breast cancer	siRNA (siFGL1) and metformin	Synergistic PD-1/PD-L1 and FGL1 pathways blockage with significant immune responses	[87]
Zeolitic imidazolate metal–organic framework	Leukemia	Nivolumab	Higher T cell activation than using free drug	[84]
Chitosan/12-mer peptide-coated gold nanorods		-	Modify m6A mRNA methylation and induce significant antitumor immunity. Combination with anti-PDL1 inhibitors inhibited cell growth and regression	[88]
Targeted PLGA and DSPE-PEG/DPPC-based nanoparticles		Tin mesoporphyrin (heme oxygenase 1 inhibitor)	Bone marrow CD11b + myeloid cells reprogramming and potent immune responses. Combination with daunorubicin suppressed cell growth	[89]
Nanoconjugates system (1F5 anti-CD20 fab – MORF1 and HPMA – MORF2)		Drug free	CD20 crosslinking and B-cell apoptosis induction. Robust cytotoxic response. Extended to treat lymphomas [91, 92]	[90]
PCL-PEG-PCL/DOTAP-based hybrid polymersomes	Lymphoma	OVA antigen and MPLA and imiquimod adjuvants (cancer vaccine)	Sustained release and internalization of antigen with cytokine production and antigen cross-presentation. Amplified immune response. Prophylactic effect with tumor growth reduction	[81, 82]
Nanocomplexes (molecular vaccines – Evans blue)		Antigen and CpG adjuvant (cancer vaccine)	Self-assembled in vivo with endogenous albumin. Potent immune responses and tumor growth inhibition	[93]
Engineered leukocyte membrane (with peptide-loaded MHC-1 and anti-CD28)-coated superparamagnetic nanoclusters		-	Synthetic antigen presenting cells. Tumor growth reduction by antigen-specific cytotoxic T cell expansion and stimulation. No systemic toxicity	[94]
Sodium alginate-coated DOTAP-based liposomes		mRNA	Enhanced transfection efficiency. Lysosome escape capacity. Induction of OVA-specific CTL proliferation and antitumor effects	[95]

**Fig. 3:**
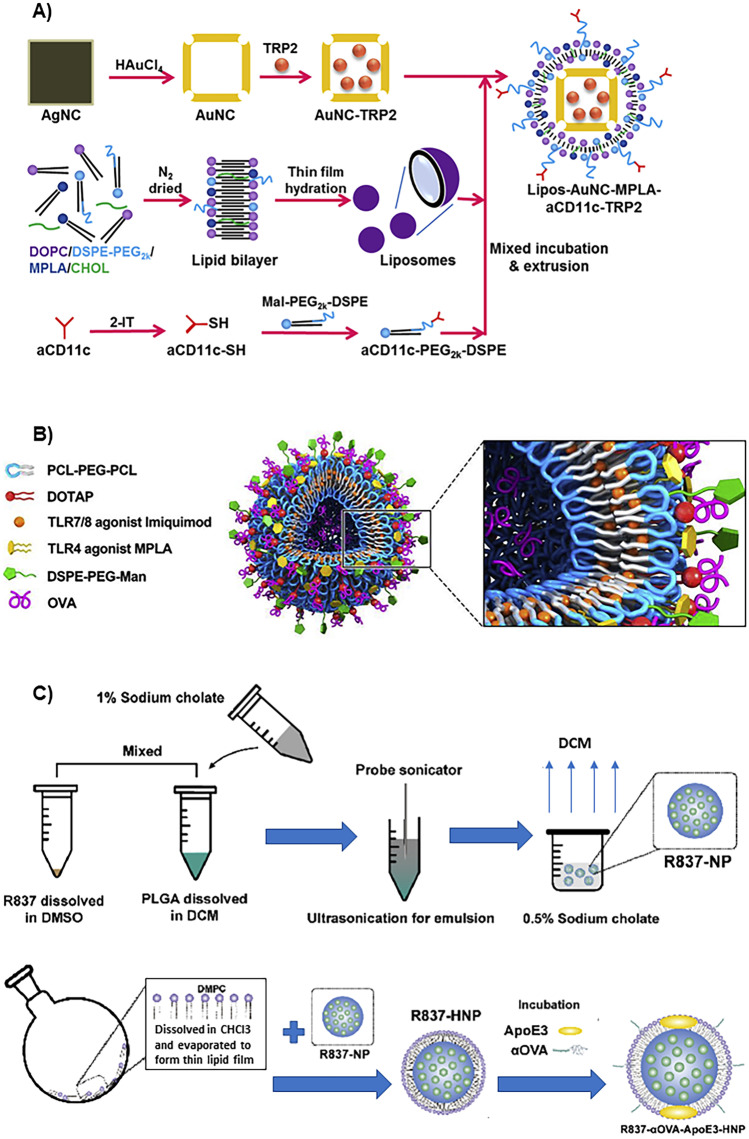
Examples of some hybrid structures that are able to accommodate all the components of cancer vaccines in the same vehicle, using the different composition of its core and the layers of its structure. **A** Synthesis procedure of gold nanocages coated with liposomes to deliver a melanoma antigen and the adjuvant MPLA to DCs. The antigen is loaded in the hollow core of the gold nanocages, and the adjuvant is incorporated in the liposomal coating that allows the conjugation of the targeting moiety. Reprinted from reference [80] with permission. **B** Hybrid polymersomes based on PCL-PEG-PCL and DOTAP that includes the OVA antigen (in the inner PEG cavity and the DOTAP coating), and two adjuvants, imiquimod (in the PCL membrane) and MPLA (in the DOTAP coating). Reprinted from reference [81] with permission. **C** Synthesis procedure of hybrid nanovaccines composed of a core of PLGA that includes the adjuvant, imiquimod, and a lipid membrane of DMPC that incorporates the antigen (αOVA) and the targeting ligand, apolipoprotein E3. Reprinted with major modifications from reference [82] with permission

The use of hybrid nanovaccines permits the co-delivery of specific antigens, along with adjuvants and targeting ligands, to DCs, in the same vehicle, using the different composition of the core and the layers of its structure. Figure 3 shows some examples of the usefulness of this hybrid structure to accommodate all the components of cancer vaccines, which are described below in the text. Liang et al. synthesized gold nanocages (AuNCs) coated with liposomes to deliver a specific melanoma antigen derived from the tyrosinase-related protein 2 (TRP2) and the adjuvant MPLA to DCs so that they process the antigen and activate CD8 + T lymphocytes, generating an immune response that inhibits tumor growth and metastasis in B16-F10 melanoma animal models. In this formulation, AuNCs provide a hollow core, which is perfect for loading drugs like the TRP2-based antigen, a controllable structure with a surface that can be modified, and exceptional optical properties that permit their in vivo tracking by fluorescence and photoacoustic imaging. Liposome coating adds biocompatibility and stability to the formulation, protects and controls the release of the encapsulated antigen in the AuNCs, facilitates the incorporation of the hydrophobic adjuvant (MPLA), and permits the facile conjugation of the targeting moiety to reach the DCs, the CD11c antibody (Fig. 3A) [80].

Similarly, the group of Zhang synthesized hybrid polymersomes using poly(ε-caprolactone)-PEG-poly(ε-caprolactone) (PCL-PEG-PCL) and DOTAP to accomplish the co-delivery of ovalbumin and two adjuvants, MPLA and imiquimod. Their hybrid structure permits the direct incorporation of the antigen in the inner hydrophilic PEG cavity and their electrostatic binding to the cationic DOTAP coating to get a sustained antigen release. Also, this design allows the encapsulation of both adjuvants in the hydrophobic PCL membrane (imiquimod) and in the lipid DOTAP coating (MPLA), in such a way they can interact with the TLR7-8 and TLR4 of DCs and generate an amplified immune response (Fig. 3B). The cationic lipidic coating facilitated antigen internalization in DCs, generating cytokines and a significant antigen cross-presentation that initiated humoral immune responses in mice. The prophylactic effect of this formulation in decreasing tumor appearance and growth after EG7-OVA cancer cell administration was demonstrated in mice [81, 83].

Likewise, Zhou et al. presented a hybrid nanovaccine, whose synthesis procedure is shown in Fig. 3C, that was composed of a core of PLGA that includes the adjuvant, imiquimod, and a biomimetic lipid membrane of 1,2-dimyristoyl-sn-glycero-3-phosphocholine (DMPC) that coats the surface of the polymeric core and incorporates the model antigen (αOVA) and the targeting ligand, apolipoprotein E3, which facilitates the nanosystem uptake into DCs by macropinocytosis. The formulation produced a significant antigen presentation that activated CD8 + T lymphocytes, a prophylactic effect in preventing metastasis, and a high therapeutic effect in combination with checkpoint inhibitors in mice bearing B16-OVA tumor cells [82].

In order to overcome the low response rate of immune checkpoint inhibitors in the clinical practice, Alsaiari et al. reported the encapsulation of nivolumab into metal–organic frameworks, based on the coordination of metal ions (Zn^2+^) with organic ligands (2-methylimidazole). These hybrid structures possess high porosity and biocompatibility, excellent loading capacity and stability, and show tunable and pH-dependent release properties. The authors demonstrated the superior capacity of this nivolumab-loaded zeolitic imidazolate framework to activate T cells in acute myeloid and chronic lymphoid leukemias when compared to the free drug. Additionally, the hybrid nanocarriers were coated with MCF-7 membranes to deliver nivolumab to the tumor micro-environment and treat 4T1 tumor-bearing mice [84]. Similarly, Ou et al. proposed hybrid PLGA/DSPE-PEG nanoparticles to deliver the cytotoxic imatinib to Treg cells and downregulate them. The PLGA core of the system permitted to encapsulate the poorly soluble imatinib and avoid its cytotoxicity on normal cells, and the DSPE-PEG external layer allowed to conjugate a tLyp1 peptide to target the Treg cells in the tumor microenvironment, controlled the drug release, and provided biomimetic properties that increased tumor accumulation. The combination of this hybrid platform with an anti-CTLA-4 antibody generated significant immune responses and antitumor effects in a B16/BL6 melanoma tumor model [85].

Hybrid nanocarriers are also being utilized in gene therapy-based cancer immunotherapy. For example, He et al. synthesized hybrid nanoparticles composed of mannosylated carboxymethyl chitosan, protamine sulfate, and calcium carbonate to transport CpG oligodeoxynucleotides to macrophages in order to modulate their polarity towards the M1 phenotype with antitumor activity through their interaction with TLR9, resulting in high levels of proinflammatory cytokines in RAW264.7 cells. Although free oligodeoxynucleotides show little or no penetration through cell membranes, their inclusion within this hybrid structure allowed them to reach macrophages by the mannose moiety of the carboxymethyl chitosan, increased their cell uptake due to the protamine sulfate, and produced their pH-sensitive intracellular release as a result of their inclusion into calcium carbonate [86]. Biomimetic hybrid nanoplatforms have been used to combine gene therapy immunotherapies with PD-1/PD-L1 blockage. In this regard, Gong et al. used PLGA nanoparticles to co-encapsulate siRNA that silence the fibrinogen-like protein 1 (FGL1) gene and metformin (Met), which can degrade PD-L1 and generate other antitumor effects. The siFGL1/Met-loaded PLGA NPs were coated with a biomimetic membrane composed of membranes of RAW264.7 macrophages and 4T1 cancer cells to provide the system with multi-targeting ability. This hybrid platform generated synergistic in vivo effects against breast cancer by blocking the PD-1/PD-L1 and FGL1 pathways with significant immune responses [87].

Nevertheless, a large number of studies can be found in the literature about the use of these hybrid systems in combined cancer therapies, merging immunotherapy with chemotherapy, photodynamic therapy, radiotherapy, and/or photothermal therapy, among others, resulting in impressive outcomes. However, once again, their clinical translation can be challenging due to the complexity of the systems, being critical to the optimization of the release profiles and timing of each drug type.

#### Using hybrid nanostructures for immunotherapy delivery in hematological cancers

Focusing on the particular case of hematological cancers, there are very few examples of the use of hybrid-based carriers to overcome the problems of the immunotherapy-based therapies, which are also collected in Table 5.

Regarding leukemia, to the best of our knowledge, apart from the study of Alsaiari et al. [84], there are only other two studies that used hybrid structures for AML immunotherapy. Du et al. demonstrated the capacity of gold nanorods to modify m^6^A mRNA methylation and induce significant antitumor immunity in AML. In order to improve their biodistribution and add active targeting capacities, the authors functionalized the surface of the gold nanorods with chitosan and a 12-mer peptide. This hybrid structure increased the efficacy of PD-1/PD-L1 inhibitors, and their combination with anti-PDL1 antibodies inhibited AML cell growth and regression in leukemia-bearing mice, which significantly increased their survival [88]. Besides, Yong et al. delivered a heme oxygenase 1 inhibitor (tin mesoporphyrin) by means of lipid/polymer hybrid nanoparticles to reprogram bone marrow CD11b + myeloid cells and trigger significant immune responses. The polymeric PLGA core of the formulation permitted the encapsulation of the hydrophobic inhibitor and the lipidic shell, which was composed of DSPE-PEG and DPPC (1,2-dipalmitoyl-sn-glycero-3-phosphocholine), increased cellular internalization, and allowed the functionalization of the carrier for AML cell targeting. Moreover, the heme oxygenase 1 inhibition avoided the resistance to chemotherapeutic drugs and the combination of this hybrid formulation with daunorubicin suppressed leukemia cell growth in AML-bearing mice [89].

Moreover, the group of Kopeček developed “drug-free macromolecular nanotherapeutics” that produced the crosslinking of the CD20 antigens in the surface of chronic lymphocytic leukemia (CLL) cells for apoptosis induction. The system combined biological and synthetic materials and was composed of two nanoconjugates, a Fab’ fragment of the 1F5 anti-CD20 conjugated with a MORF1 (morpholino-1) oligonucleotide and a N-(2-hydroxypropyl)methacrylamide (HPMA) copolymer grafted with the MORF2 oligonucleotide. The MORF1-MORF2 hybridization induced CD20 crosslinking and B-cell apoptosis in CLL cells from patients and generated a significantly more robust cytotoxic response than that produced by the existing anti-CD20 therapies. The authors extended this approach to the treatment of other B-cell malignancies, such as some of the more predominant Non-Hodgkin lymphomas [90–92].

In the case of lymphoma, apart from the works of the group of Zhang [81, 83] and those of the group of Kopeček [91, 92], there are only three studies about the utilization of combined materials for lymphoma treatment using immunotherapy to the best of our knowledge. Zhu et al. used albumin-binding vaccines, which were obtained from conjugating molecular vaccines with Evans blue, to obtain nanocomplexes that self-assembled in vivo with endogenous albumin to deliver an antigen and an adjuvant (CpG) to lymph nodes. This hybrid platform generated potent immune responses and inhibited tumor growth in EG7-OVA, B16F10, and MC38 animal models [93]. Zhang et al. developed synthetic antigen-presenting cells (sAPCs) by means of a core–shell nanostructure composed of a core of superparamagnetic nanoclusters and a coating of engineered leukocyte membranes that incorporated peptide-loaded MHC-1 and anti-CD28 for targeting purposes. The sAPCs were able to reduce tumor growth in EG7 tumor-bearing mice without systemic toxicity by antigen-specific cytotoxic T cell (CTL) expansion and stimulation [94]. Duan et al. reported that the inclusion of a sodium alginate coating into DOTAP liposome-based mRNA nanovaccines enhanced the transfection efficiency of liposome-mRNA complexes because of the modification of the endocytosis pathway and the appearance of a lysosome escape capacity. This new hybrid mRNA nanovaccine significantly induced OVA-specific CTL proliferation and antitumor effects in EG7-OVA tumor-bearing mice [95].

However, to date, no studies have been found on the use of hybrid nanostructures for multiple myeloma immunotherapy to the best of our knowledge.

### Hydrogels

Hydrogels, which are three-dimensional hydrophilic polymeric networks able to imbibe large amounts of water or biological fluids, resemble natural living tissue more than any other class of synthetic biomaterials due to their soft consistency and high-water contents that contributes to their biocompatibility. Due to the multitude of materials that can be utilized, hydrogels are an ideal method of drug delivery in the field of cancer immunotherapy. The materials can be determined by the site of implantation, release kinetics, biocompatibility, and immunogenicity. Additionally, research suggests that the usage of hydrogels could actively regulate the kinetics of the immune response [96]. Hydrogels are often needed for the delivery of biologics, such as large proteins, since these therapeutics will not be able to travel through small pores and require the gel structure. Nevertheless, hydrogels do have some disadvantages, such as their tendency to attract proteins from the surrounding environment which can lead to fouling of the material; but that is a small penalty to pay for the use of hydrogels allowing the controlled release of larger therapeutics.

Hydrogels have been used to deliver multiple types of immunotherapies based on peptides, proteins, nucleic acids, and cells. Tables 6, 7, 8, and 9 include some examples of research on these hydrogel systems for immunotherapy delivery. These works are further described throughout this section. For each of these systems, the possibility of transplantation, release kinetics, and biocompatibility/immunogenicity vary and are discussed. Implantation should be done with subjects with a characteristic length of up to 1 cm. The larger the system, the more difficult it will be to control the release behavior, as larger systems have one surface area through which it is difficult to control the system. Release kinetics should generally be on the order of around 30 min or smaller, although there are some cases in which an extended release is preferred. Release kinetics do not have to be a constant release but can be a situation where some burst release occurs that tapers off to a lower value. Furthermore, the biocompatibility of the system is a must.

**Table 6:** Examples of hydrogel systems used to deliver immunotherapies based on peptides and proteins

**Hydrogel system**	**Cancer treated**	**Therapeutic delivered**	**Main results**	**Ref**
Bio reducible cationic alginate-polyethylenimine nanohydrogel	Melanoma	OVA Proteins	Bioreducibility of the nanogels increased cell-mediated tumor cell lysis	[98]
Amphiphilic pH-sensitive galactosyl dextran-retinal nanohydrogel	Melanoma	OVA Proteins	Promoted DC maturation by activating RAR signaling, facilitated antigen uptake, cytosolic antigen release, and enhanced MHC I antigen presentation	[99]
Thermosensitive hydrogels from poly(ethylene glycol)-poly(γ-ethyl-L-glutamate) diblock copolymers	Melanoma	Interleukin-15 (IL-15) and cisplatin (CDDP)	Codelivery of IL-15 and CDDP suppressed tumor growth rate. Hydrogels degraded rapidly, indicating biocompatibility	[100]
Peptide-based hydrogels from melittin-(RADA)n	Malignant ascites, leukemia	Specific Ca^2+^/calmodulin-dependent protein kinase II (CAMKII) inhibitor, KN93	Controlled release of KN93. Antitumor properties in a mouse model of malignant ascites. Stabilizes classical oncoprotein c-Myc levels in leukemia carcinomas	[101]
Nanofibrous hydrogel based on the supramolecular co-assembly of antigen epitope-conjugated peptides	Lymphoma	Peptide antigens targeting CD8 or CD4 T cell receptors	Increase in T cell immunity and stimulation of CD8 and CD4 T cells	[102]
DNA-based hydrogel	Lymphoma	Cationic protein/peptide antigens	Controlled release of antigens. Reduction of tumor growth and significant antitumor effects	[103]
Cholesterol-modified DNA-based hydrogel	Lymphoma	Denaturalized antigen	Delayed release of antigen. Potent cancer immunity	[104]
Injectable in situ crosslinked dextran-based hydrogel	Lymphoma	Recombinant human interleukin-2 (rhIL-2)	Same therapeutic efficacy that using free rhIL-2, but with a single treatment	[105]
Injectable in situ–forming PEG-poly(L-lactide)/PEG–poly(D-lactide)-based hydrogel	Lymphoma	Recombinant human interleukin-2 (rhIL-2)	Comparable therapeutic efficacy that using free rhIL-2, but delayed in time	[106]

**Table 7:** Examples of hydrogel systems used to deliver immunotherapies based on nucleic acids

**Hydrogel system**	**Cancer treated**	**Therapeutic delivered**	**Main results**	**Ref**
DNA polyaptamer hydrogel (PAH) that can be cut by Cas9/sgRNA	Melanoma	PD-1 DNA aptamer	Increased immune cell infiltration, remained at the injection site the longest, and had the largest antitumor effect as compared to the free aptamer or the hydrogel	[107]
Thermosensitive hydrogel composed of chitosan, N-[(2-hydroxy-3-trimethyl-ammonium) propyl] chitosan chloride, and glycerophosphate	Pancreatic ductal adenocarcinoma (PDAC)	IRF5 mRNA/CCL5 siRNA-loaded nanoparticle complexes	Sustained release of the nanoparticle complex which upregulated IRF5 expression and down-regulated CCL5 expression in tumors. Potent antitumor responses	[108]
Nano-hydrogel self-assembled into an RNA-triple-helix hydrogel	Triple negative breast cancer	miRNA	Inhibition of triple negative breast cancer cell proliferation and migration. Hydrogels can be formed from therapeutics	[109]
Self-assembled DNA hydrogel	Lymphoma	Hexapod-like structured DNA	Photothermal immunotherapy with a significant inhibition of the tumor growth in mice	[110]

**Table 8:** Examples of hydrogel systems used to deliver immunotherapies based on cells

**Hydrogel system**	**Cancer treated**	**Therapeutic delivered**	**Main results**	**Ref**
Fibrin based in situ forming hydrogels	Glioblastoma	CAR T cells	Superior antitumor activity compared to naked CAR T cell inoculations. Live CAR T cells maintained for up to 5 days	[111]
Temperature sensitive and injectable PEG-g-chitosan hydrogel	Glioblastoma	T lymphocytes	More effective at eradicating glioblastoma than the control	[112]
Chitosan-based thermohydrogels	Melanoma	T lymphocytes	Hydrogels provided a suitable environment for encapsulation of T cells that maintained their cytotoxic function	[113]
HA-based scaffold	Leukemia/Breast cancer	EGFR-CAR NK cells	Effective ex vivo EGFR-CAR-NK cell expansion. Their administration in a leukemia model produced an increased survival rate. Their inclusion in the scaffold and implantation in a breast cancer model reduced recurrence and metastasis	[115]

**Table 9:** Examples of hydrogel systems used to deliver combined immunotherapies

**Hydrogel system**	**Cancer treated**	**Therapeutic delivered**	**Main results**	**Ref**
Self-assembled PEG-b-poly(lactic acid) nanoparticles and dodecyl-modified hydroxypropyl methylcellulose hydrogels	Medulloblastoma	T Cells and stimulatory cytokines	Improved efficacy of the hydrogel-based treatment by forming a temporary inflammatory niche	[116]
Injectable chitosan-PEG hydrogels	Retinoblastoma	CAR T cells and IL-15	Chitosan-PEG hydrogel played a critical role to support the antitumor effect of the combination therapy	[117]
Hydrogel layer encapsulated in a porous Teflon immune-microchip system	Ovarian cancer	CAR T cells and IL-15	The combination of tumor-priming oxygen release with CAR T cells and IL-15 into a hydrogel-based system showed significant efficacy to kill cancerous cells	[120]
Gelatin-hydroxyphenyl propionic acid-based hydrogel	Lung carcinoma	Oncolytic adenovirus (co-expressing IL-12 and GM-CSF) and DCs	Increased levels of IL-12, GM-CSF, and IFN-γ, indicating an antitumor immune response	[121]
Alginate-based sponge-like macroporous cryogels	Melanoma	GM-CSF and CpG-ODN	Infiltration of DCs and anti-tumor T cell responses	[122]
PEG-b-poly(L-alanine) injectable hydrogels	Melanoma/4 T-1 tumors	GM-CSF, tumor cell lysate antigens and anti-CTLA-4/anti-PD-1	Recruitment and activation of DCs, up-regulation of IgG production, and cytokine secretion	[123]
Self-assembling RADA16 peptide nanofibrous hydrogel	Lymphoma	Dendritic cells, anti-PD-1 antibodies and tumor antigens	Recruitment of host dendritic cells and drainage of activated DCs to lymph nodes	[124]
Dextran vinylsulfone and tetra-thiolated polyethylene glycol-based hydrogels	Lymphoma	DNA antigens and IL-10 silencing siRNA	Increased CD8 + cytotoxic T cell response, increased CD4 + CTL activity, and increased tumor protection as compared to naked DNA	[125]
MA-PEG/MA-alginate-based cryogel	Leukemia	AML-associated antigens, CpG-ODN and GM-CSF	Prophylactic T cell-based effects. Therapeutic antitumor responses with chemotherapy	[126]

#### Hydrogels/scaffolds for protein/peptide-based immunotherapies

As previously described, particle-based vaccine delivery systems prevent antigen degradation, increase antigen uptake by APCs and promote DCs maturation [97]. Therefore, nanoparticle-based vaccines made from hydrogel materials are likely to be successful in acting as potent adjuvants with minimal adverse effects. In fact, Li et al. developed a bio-reducible cationic alginate-polyethyleneimine (PEI) nano-hydrogels loaded with OVA as a novel vaccine. The nano-hydrogels exhibited antigen-loading capacity and minimal cytotoxicity. In addition to facilitating antigen uptake by mouse bone marrow dendritic cells (BMDCs), the nano-hydrogels also promoted intracellular antigen degradation and cytosolic release. Compared with non-reducible nano-hydrogels, this one dramatically enhanced vaccine-induced antibody production and CD8 + T cell–mediated tumor cell lysis, which could be due to their strong capability of promoting intracellular antigen processing and cytosol release as well as MHC class I/II antigen presentation. Improved results were due to the improved delivery of OVA by the nano-hydrogels [98]. Similarly, Wang et al. incorporated OVA antigens into amphiphilic pH-sensitive galactosyl dextran-retinal (GDR) nanogels that focused on MHC class I antigen presentation of exogenous antigens and retinoic acid receptor signaling (RAR). Dextran was conjugated with retinal due to vitamin A’s ability to enhance antigen-specific antibody production, and then it was subjected to galactosylation to get DC-targeting ability. The nanohydrogels were then injected into B16-OVA-bearing mice, and no adverse effects were observed indicating biocompatibility. The results indicated that the GDR nano-hydrogel promoted DC maturation by activating RAR signaling and facilitated antigen uptake and cytosolic antigen release. In addition to this, MHC I antigen presentation was enhanced, which caused significant anti-cancer immune responses [99].

Moreover, Wu et. al. developed in situ–forming thermosensitive hydrogels synthesized from poly(ethylene glycol)-poly(γ-ethyl-L-glutamate) diblock copolymers (mPEG-b-PELG) for the co-delivery of interleukin-15 (IL-15) and cisplatin (CDDP) against melanoma. After being injected into mice inoculated with B16F0-RFP, the authors observed anticancer efficacy due to the combination of CDDP-mediated S arrest, and IL-15/CDDP-induced recovery of CD8 + T cell and NK cell populations that reduced immunosuppression and enhanced antitumor immunity. Among the test groups, the hydrogel with IL-15 and CDDP suppressed the tumor growth more efficiently. In addition to this, the mPEG-b-PELG hydrogels degraded gradually within 3 weeks of the subcutaneous injection and were biocompatible both in vitro and in vivo [100].

A majority of the hydrogel platforms that have been developed to deliver peptide/protein-based immunotherapies are for solid tumors. However, one study mentions its applicability to leukemia treatment. In this work, the authors developed peptide-based hydrogels from melittin-(RADA)n for delivery of a specific Ca^2+^/calmodulin-dependent protein kinase II (CAMKII) inhibitor, KN93, with a capacity of reprogramming tumor-associated macrophages. The study showed that the system got a controlled release of KN93 and displayed antitumor properties in a mouse model of malignant ascites with biodegradability and biocompatibility observed. This system is also applicable to leukemia as it has been shown that CAMKII stabilizes classical oncoprotein c-Myc levels in leukemia carcinomas and KN93 is capable of inhibiting CAMKII activity [101].

In addition, Su et al. developed a nanofibrous hydrogel, through the supramolecular coassembly of antigen epitope-conjugated peptides, against EG.7.OVA lymphoma tumors. These hydrogels targeted CD8 or CD4 T cell receptors. Within mice models, the authors observed an increase in T cell immunity and the stimulation of CD8 and CD4 T cells with all mice surviving and no adverse effects indicating biocompatibility. As compared to a free peptide vaccine or an aluminum-adjuvanted peptide formulation, this method resulted in a higher cancer immunotherapy efficacy [102]. Also, Umeki et al. described the use of DNA-based hydrogels for antigen delivery in lymphoma tumors. They reported the complexation of the cationic ethylenediamine-conjugated OVA with hexapod-like structured DNA/CpG-based hydrogels, which show immunostimulatory properties, to achieve a controlled release of the antigen in EG7-OVA tumor-bearing mice with a reduction of the tumor growth without adverse effects. Based on that, the authors included a cationic peptide antigen in the same formulation instead of the protein antigen, resulting in a significant antitumor efficacy in EG7-OVA tumors [103]. Moreover, the same authors achieved a potent cancer immunity in EG7-OVA lymphoma-bearing mice by delaying even more the antigen release. This was accomplished by modifying the DNA with cholesterol and denaturalizing OVA with urea, obtaining a stronger hydrophobic interaction between both [104].

There is also a fairly old study that describes the therapeutic efficacy of a rhIL-2-loaded dextran-based hydrogel to treat local SL2 lymphomas. The hydrogel was liquid at the time of injection and in situ gelled by physical crosslinking at physiological temperature and pH, which permits a controlled release of the protein for 5 days and the preservation of its structure and biological activity. The material used did not show inflammatory reactions and was replaced by fibroblast over time. In vivo the therapeutic efficacy of this system was at least comparable to that achieved with the same dose of free rhIL-2, but using a single treatment [105]. Similarly, Hiemstra et al. tested the therapeutic efficacy of another rhIL-2-loaded in situ–forming hydrogel in SL2-lymphoma-bearing mice, this time using PEG-poly(L-lactide) and PEG–poly(D-lactide)-based copolymers. As in the case of the dextran-based hydrogel, the effect of this formulation was comparable to that of the free protein, but delayed in time because of the controlled release process. In addition to this, the system was biodegradable under physiological conditions indicating biocompatibility [106].

Table 6 summarizes the main aspects of the studies described in this section that use hydrogels to deliver immunotherapies based on peptides and proteins.

#### Hydrogels/scaffolds for nucleic acid–based immunotherapies

Aside from proteins/peptides, nucleic acids can also be loaded in hydrogels for the purpose of immunomodulation. Lee et al. developed a DNA polyaptamer hydrogel (PAH) that can be cut by Cas9/sgRNA for the programmed release of a PD-1 DNA aptamer that can block the interaction between the PD-1 cell-surface and the PD-L1 tumor cell-surface. DNA aptamers are advantageous over antibodies as they exhibit higher thermal stability and lower immunogenicity. The in vitro Cas9-edited cleavage of PAH was completed within three days. The in vivo interaction of PD-1 with PD-L1 was imitated by culturing splenocytes on PD-L1-precoated plates. These authors observed an increase in the secretion of IL-2 from splenocytes treated with PAH and Cas9/sgRNA compared to those treated with the free aptamer or the hydrogel only. Furthermore, in tumor-bearing mice, the PAH-Cas9/sgRNA system had an increased immune cell infiltration and the largest antitumor effect in melanoma and were able to be retained at the injection site for up to a few days [107]. However, the system could still be improved by increasing both the cleavage time and retention time to longer than a few days to decrease the need for repeated treatments. Furthermore, Cas9 was used as a component of the system and has possible immunogenicity that needs to be addressed.

In a different approach, Gao et al. used RNAs for cancer immunotherapy, but with a focus on treatment of pancreatic ductal adenocarcinoma (PDAC) and reprogramming the antitumoral immune niche. They developed an in situ injectable thermosensitive hydrogel composed of chitosan, N-[(2-hydroxy-3-trimethylammonium) propyl] chitosan chloride and glycerophosphate, which was loaded with lipid-immune regulatory factor 5 (IRF5) mRNA/C–C chemokine ligand 5 (CCL5) siRNA nanoparticle complexes. The co-delivery of IRF5 mRNA and CCL5 siRNA allows for the upregulation of IRF5 and downregulation of CCL5, which can help contribute to a significant increase in M1 phenotype macrophages, which can help reduce tumor growth as M1 macrophages can initiate T cell-mediated immune responses. The nanoparticles were released for up to 16 days from the hydrogel, indicating that sustained release was achieved. Indeed, when the hydrogels were injected into mice tumor tissue, a sustained release of the nanoparticle complex was observed, which upregulated IRF5 expression and down-regulated CCL5 expression in tumors. The results of this article indicated that the combination of a chitosan-based hydrogel and IRF5/CCL5-loaded nanoparticle complexes did induce potent antitumor responses in PDAC [108]. H&E staining of major organs also revealed that the hydrogel/nanoparticle complex was non-toxic to mice.

Although siRNA and microRNA (miRNA) used for RNA interference are typically delivered using cationic nanocarriers as they must be delivered intracellularly, cationic materials are typically associated with relatively high cytotoxicity. To mitigate this cytotoxicity and deliver miRNA for the treatment of triple-negative breast cancer, Ding et al. developed nano-hydrogel carriers from self-assembled DNA/RNA. The RNA hydrogel consisted of miRNA-205, a tumor suppressor, and miRNA-221, an oncomiR inhibitor, and was self-assembled into an RNA-triple-helix hydrogel by incorporating polymerized RNA transcripts of the miRNAs through Watson–Crick pairing and Hoogsteen hydrogen binding. siRNA duplexes of CXCR4 were embedded into the RNA hydrogel to block breast cancer cell metastasis, and LXL-DNA aptamer was conjugated to target triple-negative breast cancer cells. The authors showed the ability of this hydrogel to inhibit cancer cell proliferation and migration [109]. In vivo experiments in an orthotopic breast cancer mouse model showed no signs of inflammation and no body weight changes, suggesting the hydrogels were biocompatible. These experiments also showed that the tumors of mice treated with RNA hydrogels had inhibited tumor progression compared to the controls. This approach shows that hydrogel materials can be self-assembled from the therapeutics themselves, which may provide unique applications in the future as it can reduce side effects associated with materials.

Hydrogels synthesized from nucleic acids can also be combined with other materials to create more successful treatments. The combination of hydrogels with inorganic-based nanocarriers allows for the successful use of photothermal immunotherapy in cancer. In this regard, Yata et al. synthesized a self-assembled DNA hydrogel that included hexapod-like structured DNA, CpG sequences, and gold nanospheres and nanorods, which are photothermally active and able to release highly ordered DNAs. The DNA utilized here contained CpG sequences, which is a well-known ligand for TLR9, to increase cytokine release from TLR9-positive cells. Laser irradiation of the hydrogel resulted in the release of the DNA, thus allowing for triggerable release for up to 4 min. This was the fastest release of all delivery platforms used to deliver nucleic acids, in addition to the only one that is triggerable. The triggerable release is useful for targeted release as the user can choose to trigger release only once the therapy reaches the tumor site. Significant cytotoxicity was not observed in cell experiments. This formulation was tested in EG7-OVA tumor-bearing mice, observing photothermal immunotherapy when irradiated with laser, which induced a significant inhibition of the tumor growth in the animals [110].

Table 7 summarizes the main aspects of the studies described in this section that use hydrogels to deliver immunotherapies based on nucleic acids. The approaches reviewed here are all working to alter the immune system in different ways. While all approaches appear to be viable, it is difficult to compare them as they work by different mechanisms. Many of these could be employed or even combined to treat the myriad of cancers that are currently incurable. The approach by Lee et al. [107] utilizes DNA aptamers in a self-assembled hydrogel to block the PD-1/PD-L1 immune checkpoint pathway. The approach by Gao et al. [108] uses the co-delivery of mRNA and siRNA with a combined nanoparticle/hydrogel carrier to stimulate macrophages towards an M1 phenotype, thus triggering a more robust T cell-mediated immune response against the tumor. The study by Ding et al. [109] utilized hydrogel nanoparticles that were composed of self-assembled miRNA with loaded siRNA and a conjugated DNA aptamer, all with various goals of inhibiting tumor progression in triple-negative breast cancer. The last study by Yata et al. [110] synthesized hydrogels from DNA that contained CpG sequences to stimulate cytokine release and bolster the immune response; in addition, the hydrogels contained gold nanoparticles and nanorods that allowed for the triggerable release of the DNA and heat production upon irradiation with a laser. This was the only study that showed triggerable release, which is useful as it will allow for more targeted and specific delivery. Self-assembly using the RNA/DNA themselves was a trend to synthesize the hydrogels reviewed in this section, which is beneficial as it reduces the use of foreign materials introduced to the body.

#### Hydrogels/scaffolds for cell-based immunotherapies

**Fig. 4:**
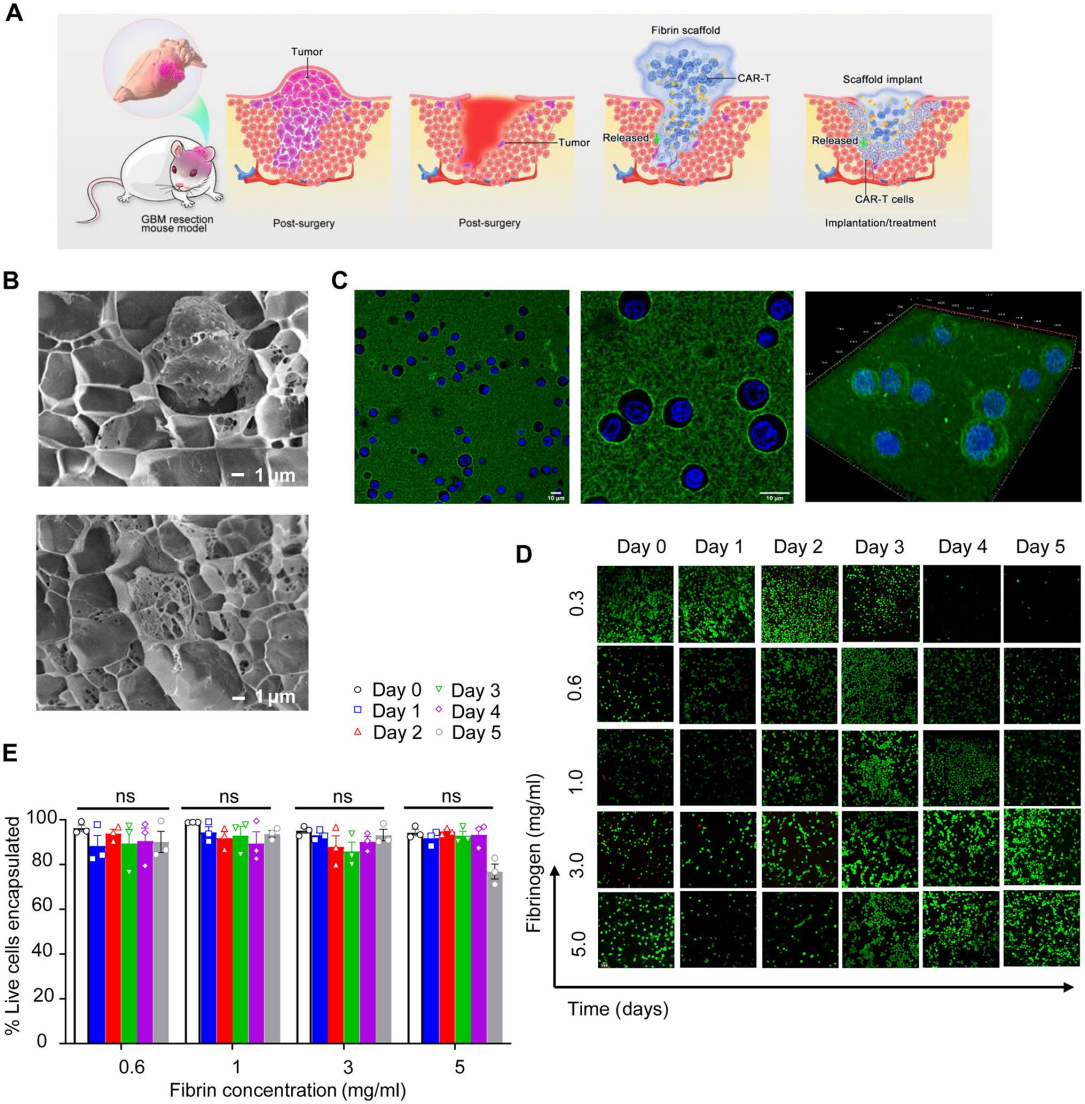
CAR T cell-loaded biomimetic fibrin-based hydrogels reported by Ogunnaike et al. that enables the controlled release of CAR T cells and sustain their viability, achieving superior antitumor activity compared to naked CAR T cell inoculations. **A** Implementation of the hydrogel into the tumor resection cavity. **B** Cryo-SEM imaging of the CAR-T cells-loaded fibrin-based hydrogel. **C** Confocal imaging of the CAR-T cells-loaded fibrin-based hydrogel (CAR T cells labelled with green fluorescein). **D** Confocal imaging of live/dead CAR T cells in the hydrogel for 5 days (live cells were labelled with green fluorescein; dead cells were labelled with red fluorescein). **E** Quantification of live CAR T cells in the hydrogel for 5 days, using different concentrations of fibrin. No significant differences were found. Reprinted from reference [111] with permission

The local delivery of CAR T cells by hydrogels into the brain may represent a clear alternative to their intravenous administration in order to avoid having to deal with the blood–brain barrier. In this regard, a study by Ogunnaike et al. sought to encapsulate CAR T cells into fibrin-based in situ–forming hydrogels for placement into the tumor resection cavity in a glioblastoma model. This was shown to be advantageous and achieve superior antitumor activity compared to naked CAR T cell inoculations, as the gel could accommodate the controlled release of CAR T cells and sustain their viability. The authors selected fibrin as the material of choice for the hydrogel as it is a natural material involved in the coagulation cascade, has similar mechanical properties to brain tissue, and is biodegradable and non-inflammatory [111]. Figure 4 shows how these scaffolds were implemented (A), their characterization (B-C), and the evidence that the hydrogel maintains the CAR T cells live for up to 5 days (D–E).

Moreover, a study looking at the use of hydrogels as a depot for T cells for glioblastoma immunotherapy was performed by Tsao et al. that used as material poly(ethylene glycol)-g-chitosan. This material is temperature-sensitive, biocompatible, biodegradable, has a low immunogenicity, and forms a solution from a gel at ~ 32 °C, thus allowing for an injectable hydrogel material. The mesh size of the hydrogel material was optimized for T cell diffusion and was shown to be more effective at killing glioblastoma than the control [112]. Another study also utilized chitosan-based hydrogels for the delivery of T cells, again to leverage the solution to gelling properties of chitosan. This study utilized sodium hydrogen carbonate and phosphate buffer as gelling agents, developing biocompatible hydrogels with appropriate macroporosity and gelation rates that provided a suitable environment for the encapsulation of viable T lymphocytes that maintained their cytotoxic function [113].

The approaches described thus far are all looking at using hydrogels for solid tumors. For hematological cancers, cell therapies have already shown to be quite effective without using carriers; however, hydrogel nanoparticles such as those developed by the group of Peppas et al. [114] could be adapted for use in leukemia and lymphoma and help improve cell therapy by increasing specificity and targeting cancer cells. In addition, scaffolds can be used as 3D-culture niches for effective ex vivo CAR T/NK cell expansion. In fact, Ahn et al. engineered a biodegradable and biocompatible 3D scaffold based on hyaluronic acid (HA) for NK-92 and EGFR-CAR-NK cell expansion, which produced significant antitumor effects. The administration of the expanded EGFR-CAR-NK cells in K562 leukemia-bearing mice resulted in an increased survival rate. The implantation of EGFR-CAR-NK-cells-loaded HA-based scaffolds in a MDA-MB-231 animal model after a partial resection of the tumor resulted in a reduction of recurrence and metastasis in the animals [115].

Table 8 summarizes the main aspects of the studies described in this section that use hydrogels to deliver immunotherapies based on cells. Furthermore, the approaches described in this section have been combined with the co-delivery of cytokines or other immunostimulatory molecules and are described in the next subsection.

#### Hydrogels/scaffolds for combination immunotherapies

**Fig. 5:**
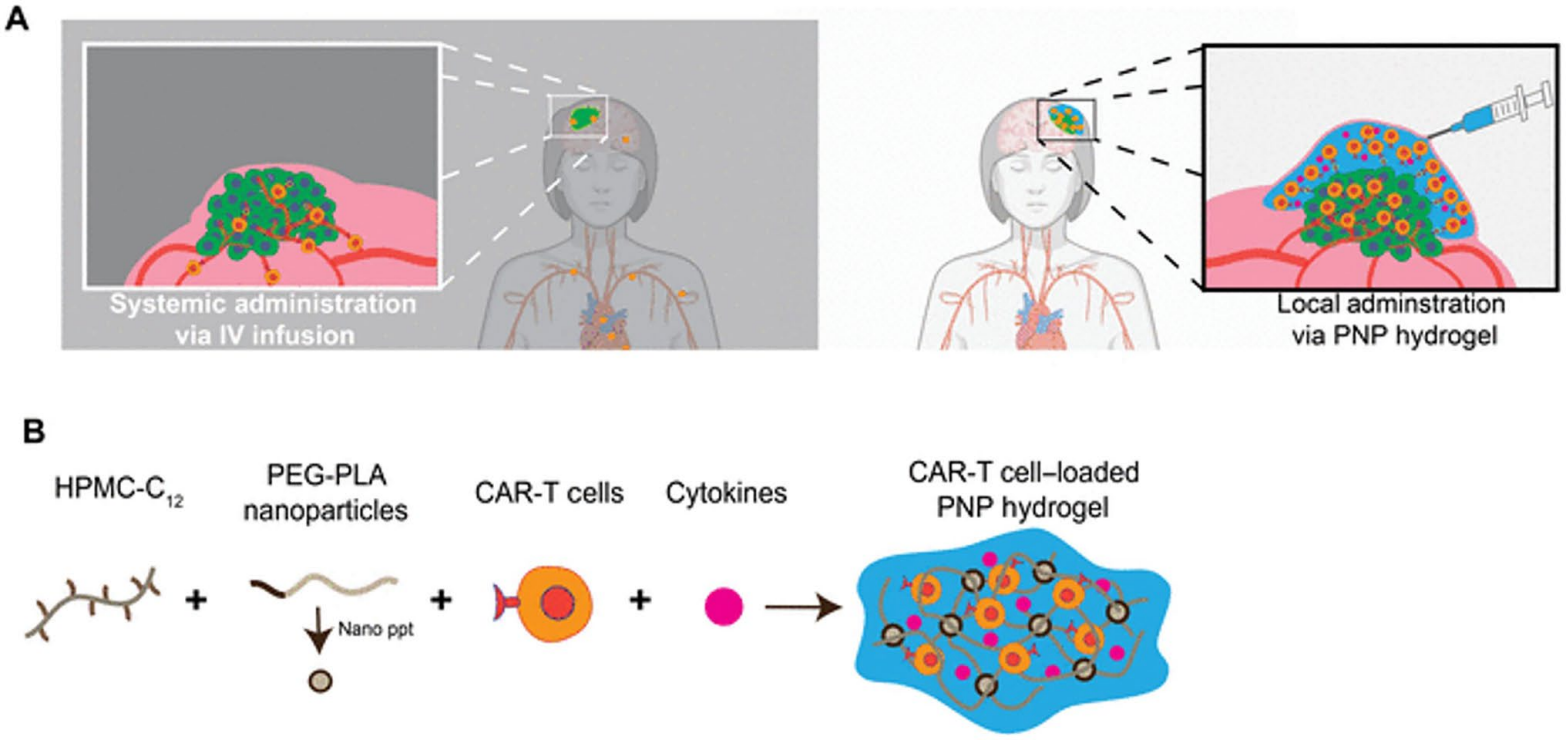
Injectable self-assembled PEG-PLA/HPMC-C_12_-based hydrogel reported by Grosskopf et al. that permits the direct administration of CAR T cells in combination with cytokines in the brain, creating a temporary inflammatory niche. **A** Comparison between the local administration of the combined immunotherapy by using the hydrogel and the conventional intravenous administration. **B** Synthesis of the PEG-PLA/HPMC-C_12_-based hydrogels that include the CAR T cells and the cytokines. Reprinted with modifications from reference [116] with permission

A promising approach for cancer immunotherapy is combining different therapies into one delivery method that can leverage advantages from the various therapy types. Delivery of immune cells with other immunostimulatory agents is a common strategy. Similar to the cell therapy studies that delivered T cells alone, one study combined T cell therapy with stimulatory cytokines, using an injectable hydrogel as the delivery vehicle for solid tumors that permits the facile administration of combined immunotherapies by direct injection, generating a temporary inflammatory niche that increases the expansion and activation of the CAR T cells (Fig. 5A). In this case, it is advantageous to deliver CAR T cells with cytokines as high cytokine concentrations are required for CAR T cell activation that can cause toxicity when delivered systemically. The hydrogel was formed by self-assembly of poly(ethylene glycol)-b-poly(lactic acid) nanoparticles and dodecyl-modified hydroxypropyl methylcellulose, which enabled the diffusion of cytokines and the active motility of the loaded CAR T cells (Fig. 5B), improving the efficacy of the treatment over the conventional intravenous strategies (Fig. 5A) [116].

Likewise, a study sought to co-deliver CAR T cells with cytokine IL-15 using biodegradable and biocompatible injectable chitosan-PEG hydrogels for the treatment of retinoblastoma. The authors showed that the combinatory approach with the hydrogel platform successfully eliminated retinoblastoma tumor cells without impairing mouse vision [117]. Previous work showed that IL-15 played a critical role in the survival of CAR T cell survival and activity [118, 119], while the current study showed the chitosan-PEG hydrogel also played a critical role to support the antitumor effect of the combination therapy [117]. Similarly, another study also sought to co-deliver CAR T cells with IL-15 using a hydrogel-based delivery system. In this case, a hydrogel layer was encapsulated in a porous Teflon immune-microchip system. The hydrogel layer, composed of hemoglobin-loaded alginate, was designed to degrade quickly, thus delivering an amount of oxygen to the tumor stroma, which is supportive of the survival of infiltrating immune cells. This led to priming the tumor for CAR T cells and IL-15, which were able to migrate from the carrier to the tumor following gel degradation. The combination of the tumor-priming oxygen release, CAR T cells, and IL-15 promoted the efficacy of the platform to kill cancerous cells [120].

Other studies seek to develop combinatorial therapies for other immune cell types, such as dendritic cells. For example, Oh et al. developed a biodegradable gelatin-hydroxyphenyl propionic acid-based hydrogel, which carried oncolytic adenovirus coexpressing interlukin-12 (IL-12) and granulocyte–macrophage colony-stimulating factor (GM-CSF), and DCs. Due to the material of their hydrogel, the therapeutics were released over a sizable time period while keeping their biological activity. When the hydrogels were injected into mice with lung carcinomas, both therapeutics were efficiently retained in the tumor tissue. Additionally, the tumors that were treated with the hydrogel were observed to have an increase in IL-12, GM-CSF, and interferon (IFN-γ) expression as compared to tumor tissue that was treated with a single treatment of adenovirus or DCs or a combination of adenovirus and DCs, which was indicative that tumors were infiltrated by CD4 + and CD8 + T cells, generating antitumor immune responses [121].

In other combinatorial approaches, researchers combine proteins and nucleic acids, rather than cells. This is because the proteins and nucleic acids can be used to stimulate or recruit immune cells to the site, which should prove to be less costly than ex vivo expansion and manipulation of immune cells, as well as avoid issues, such as the requirement to maintain the viability of biomaterial-encapsulated cells. In this regard, the Mooney group developed sponge-like macroporous cryogels from alginate to achieve the codelivery of GM-CSF, a dendritic cell enhancement factor, and cytosine-phosphodiester-guanine oligodeoxynucleotide (CpG ODN), a TLR agonist and dendritic cell-activating factor. This alginate-based gel had RGD peptides covalently coupled to promote tumor cell attachment through integrin binding. These authors reported that subcutaneously injected gels in mice were able to elicit infiltration of dendritic cells, as well as induce anti-tumor T cell responses in a melanoma model without adverse effects [122]. Similarly, another study also used biocompatible injectable hydrogels, this time synthesized from PEG-b-poly(L-alanine), to deliver GM-CSF along with tumor cell lysate antigens and checkpoint antibodies (anti-CTLA-4 and anti-PD-1). The in vivo results showed that the sustained release of the combined factors generated persistent recruitment and activation of dendritic cells, upregulation of IgG production, and cytokine secretion, being this hydrogel successful at treating melanoma and 4 T-1 tumors [123].

Regarding hematological cancers, Yang et al. sought to co-deliver DCs with anti-PD-1 antibodies and tumor antigens using a self-assembling RADA16 peptide nanofibrous hydrogel in a EG7-OVA lymphoma model. In this formulation, self-assembling peptides modulate immune responses, encapsulated antigens are ingested by dendritic cells to create dendritic cell vaccines, and anti-PD-1 are added to help alleviate tumoral immunosuppression. The injected hydrogels were able to maintain viability and maturation of the encapsulated dendritic cells, as well as promote the recruitment of host dendritic cells and drainage of activated dendritic cells to lymph nodes with no adverse effects [124]. Moreover, Singh et al. developed a synthetic immune priming center against B cell lymphoma, consisting of in situ crosslinking and chemokine-carrying hydrogel loaded with both DNA antigens and IL-10 silencing siRNA-loaded PEI-PLGA microparticles. The hydrogel attracted immature DCs at the site of administration and delivered both the DNA and siRNA to those cells. This formulation resulted in a 45% increase in CD8 + cytotoxic T cell response and a 53% increase in CD4 + CTL activity as compared to the naked DNA vaccine. Additionally, the in vivo immunization with this formulation increased protection against subsequent tumor challenges with no adverse effects. Ultimately, combining DNA antigens, chemokines, and IL-10 siRNA increases tumor protection as compared to studies delivering naked DNA as therapy [125].

In regard to leukemia, Shah et al. synthesized a macroporous cryogel based on methacrylated PEG and alginate (MA-PEG and MA-alginate) to vehiculize a combination of leukemia antigens, CpG-ODN and GM-CSF in AML-bearing mice, resulting in prophylactic T cell-based effects and significant therapeutic antitumor responses when combined with chemotherapy [126].

Table 9 summarizes the main aspects of the studies described in this section that use hydrogels to deliver combined immunotherapies. Indeed, these therapies provide opportunities for the future to develop functional cures for patients with cancer, and the drug delivery platforms that can maintain a controlled release of combined factors will be critical to the success of these therapies.

##### Conclusions and future perspectives

Using some illustrative samples, we have described the emergence of lipid, inorganic, polymeric, and hybrid-based nanocarriers and hydrogels and scaffolds in the field of cancer immunotherapy to overcome its targeting and biodistribution limitations and avoid some of its associated side effects. The very few examples identified above for the treatment of hematological cancers indicate an urgent need for more advanced studies in the field.

Nevertheless, the biggest challenge for the near future is the successful application of immunotherapy in solid tumors and the drug delivery systems will undoubtedly play an important role in this task, facilitating the entrance of the immunotherapeutic drugs into the tumor tissue by active targeting strategies or the physicochemical optimization of the carriers (morphological and surface properties). Of course, it is clear that for all this, numerous challenges need to be addressed for the clinical translation of nanomedicines. First of all, the toxicology of nanomaterials should be considered to assess their potential influence on the body due to their chemical composition and their nanoscale properties [127, 128]. In this regard, nanocarriers need to face a greater number of technical hurdles to obtain the approval by the regulatory agencies than conventional formulations.

In addition, the interaction of nanocarriers with cells is a hot topic since their internalization is normally a prerequisite for their success in improving the immunotherapeutic approaches [129].

Another challenge is the improvement of the transfection efficiency of carriers in vivo to be able to definitively substitute the ex vivo modification of T cells for the CAR technology. Undoubtedly, the future of nanocarriers in clinical immunotherapy will be their usage in combination with other immunotherapeutic approaches to get synergies since, frequently, tumors show multiple immunosuppressive pathways that must be faced with multiple approaches [130]. In this regard, nanocarrier-based nanomedicines are allowing the co-administration of different immunotherapeutics at the same time, getting improved immune responses because of their synergistic actuation [131].

Moreover, the possibility of delivering different types of drugs in the same nanovehicle is allowing the effective combination of immunotherapy with chemotherapy or radiotherapy [89, 132–134]. The nanocarriers permit the efficient spatiotemporal delivery of the different drugs included in the nanovehicle. Still, the role of biomarkers is crucial to identify patient outcomes when these approaches are applied and to detect the immunosuppressive pathways. Therefore, the field of biomarker analysis in immunotherapy must be further developed [135–137].

## Data Availability

Not applicable to this article.
